# Astroglial CB1 Cannabinoid Receptors Mediate CP 55,940-Induced Conditioned Place Aversion Through Cyclooxygenase-2 Signaling in Mice

**DOI:** 10.3389/fncel.2021.772549

**Published:** 2021-11-23

**Authors:** Jin Cong, Kangrong Lu, Wenjie Zou, Ziming Li, Zhipeng Guo, Xiangzhen Tong, Jiawei Zheng, Jianping Zhu, Shuji Li, Wangming Zhang, Yanwu Guo, Tian-Ming Gao, Rongqing Chen

**Affiliations:** ^1^Guangdong Province Key Laboratory of Psychiatric Disorders, Department of Neurobiology, School of Basic Medical Sciences, Southern Medical University, Guangzhou, China; ^2^Department of Histology and Embryology, School of Basic Sciences, Southern Medical University, Guangzhou, China; ^3^The National Key Clinic Specialty, Guangdong Provincial Key Laboratory on Brain Function Repair and Regeneration, Department of Neurosurgery, The Engineering Technology Research Center of Education Ministry of China, Zhujiang Hospital, Southern Medical University, Guangzhou, China; ^4^Key Laboratory of Mental Health of the Ministry of Education, Guangdong-Hong Kong-Macao Greater Bay Area Center for Brain Science and Brain-Inspired Intelligence, Southern Medical University, Guangzhou, China

**Keywords:** astrocyte, cannabinoid, cannabinoid receptor, conditioned place aversion, cyclooxygenase-2

## Abstract

Cannabinoids (CBs), such as phytocannabinoids, synthetic CBs, and endogenous CBs, can be neuroprotective, rewarding, or aversive. The aversive effects of CBs may hinder their medical and recreational applications. It is unknown which type of CB receptors mediates the direct aversive effects of synthetic CB CP 55,940 which is an analog of Δ^9^-tetrahydrocannabinol, the major psychoactive component of marijuana. In this study, we address this question by taking the advantage of systematic type 1 CB receptor (CB1R) knockout mice and conditional reinstatement of this receptor only in astrocytes. We show that CP 55,940 at a concentration of 1 mg/kg induces conditioned place aversion (CPA) and the CPA effect of CP 55,940 is mediated by the astroglial CB1Rs. Inhibiting cyclooxygenase-2 (COX-2) eliminates CP 55,940-induced CPA in mice that only express CB1Rs in astrocytes. These findings conclude that CPA effect of CP 55,940 is mediated by the astroglial CB1Rs through COX-2 signaling, suggesting that selective COX-2 inhibition or precise isolation of astroglial CB1R activity may be the strategy for treating aversive response of medical and recreational administrations of marijuana.

## Introduction

Marijuana is widely known as a drug of abuse. Unlike other abused drugs, marijuana produces not only psychostimulant, euphoric, and rewarding effects, but also dysphoric and aversive effects. The Δ^9^-tetrahydrocannabinol (Δ^9^-THC) is the major psychoactive component of marijuana, the actions of which can be mimicked by its synthetic analogs CP 55,940 and HU-210 or some other synthetic cannabinoids (CBs). Animal models and clinical studies revealed that the aversive effects were induced by the high doses of Δ^9^-THC (Hall, 2015; Norris et al., 2019; Spiller et al., 2019) or synthetic CBs such as CP 55,940, WIN55,212-2 (McGregor et al., 1996; Spiller et al., 2019). Besides, marijuana has been used for medical purposes since ancient times. The merit of CBs in medicine is supported by the modern biological and medical researches evidencing or suggesting benefits of CBs for various conditions, such as pain, epilepsy, anorexia, neurodegenerative and psychiatric diseases, inflammatory disorders, asthma, cancer, and autoimmune diseases (Russo, 2007; Chen et al., 2012; Fraguas-Sanchez and Torres-Suarez, 2018; Zhang and Chen, 2018; Stasiulewicz et al., 2020; Huang et al., 2021). However, the aversive effects of marijuana and other CBs may hinder their recreational and medical applications.

The main biological targets for CBs are type 1 CB receptors (CB1Rs) and type 2 CB receptors (CB2Rs) that were cloned in the 1990s (Matsuda et al., 1990; Munro et al., 1993). In the brain, CB1Rs are abundantly and widely expressed in hippocampus, amygdala, neocortex, striatum, hypothalamus, brainstem, and cerebellum (Busquets-Garcia et al., 2018). The expression of CB2Rs is most higher in the immune system, but they are also expressed widely in the brain with a relatively lower abundance (Roche and Finn, 2010). The mesolimbic and subcortical structures are primarily important for reward and aversion-related brain functions. Microinfusions of Δ^9^-THC into the anterior and posterior nucleus accumbens (NAc) differentially modulate the neuronal activity and produce reward and aversion, respectively (Norris et al., 2019). Activation of CB1Rs of the ventral tegmental area (VTA) and central amygdala (CeA) induces conditioned place preference (CPP), while that of the medial prefrontal cortex (mPFC) induces conditioned place aversion (CPA) *via* interacting with hippocampus (Navabpour et al., 2021). It was reported that CB1Rs on glutamatergic neurons are responsible for Δ^9^-THC-induced aversive effects (Han et al., 2017), and that CB1Rs of interpeduncular nucleus-innervating medial habenular neurons control aversive responses by modulating the corelease of glutamate and acetylcholine (Soria-Gomez et al., 2015). Moreover, some studies suggested a role of CB2R in mediating the aversive effects of CBs as CB2R antagonist (AM630) attenuates inhibitory action of Δ^9^-THC and WIN55,212-2 on the brain-stimulation reward (Spiller et al., 2019), while overexpression of CB2Rs decreases cocaine self-administration (Aracil-Fernandez et al., 2012). Therefore, it is still controversial regarding which type of CB receptors mediates the aversive effects of some CBs.

The both types of CB receptors are not only located in the neurons, but also in glial cells (Roche and Finn, 2010; Chen et al., 2017). In astrocytes, activation of CB1Rs can modulate the astroglial metabolism (Jimenez-Blasco et al., 2020), mediate neuron-astrocyte communication (Navarrete and Araque, 2008) and cause synaptic and memory impairments (Chen et al., 2013). Besides, as a powerful regulator of inflammation, astrocytes activated by the activation of CB1Rs can modulate the neuroinflammation through inflammatory factors such as interleukin-1 (IL-1) (Fields et al., 2020), cyclooxygenase-2 (COX-2) (Zhang and Chen, 2008). It is unknown whether astroglial CB receptors participate in the aversive effects of CBs. To analyze the type of CB receptors and cellular mechanism underlying the aversive effects of CBs, we used mouse model of CPP induced by the synthetic CB CP 55,940 and genetically modified the expression of CB1Rs in the present study. We found that astroglial CB1 receptors mediate aversive effect of CP 55,940 which can be eliminated by inhibiting the COX-2 signaling.

## Results

### High Dose of Synthetic Cannabinoid CP 55,940 Induces Conditioned Place Aversion

**FIGURE 1:**
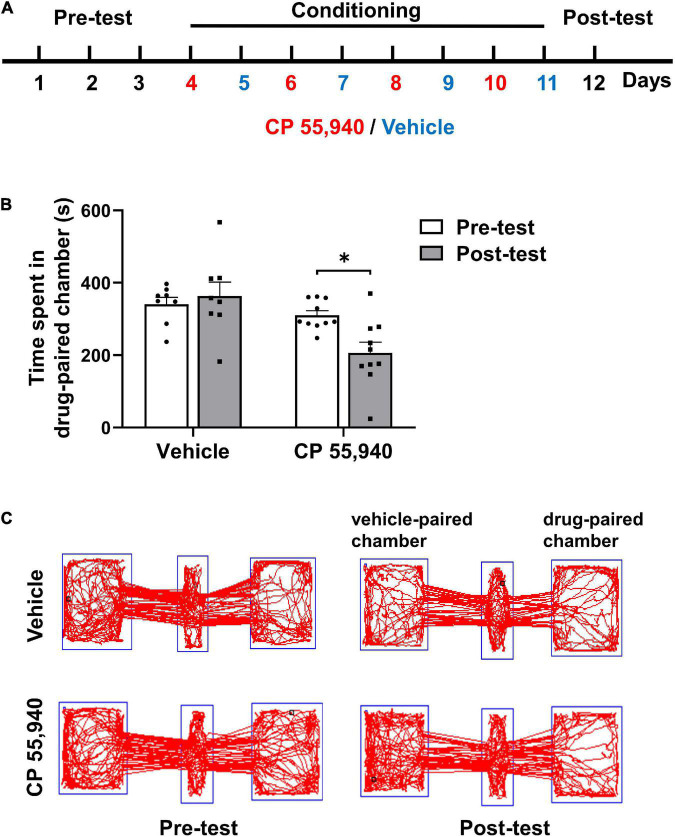
CP 55,940 at a dose of 1 mg/kg induces conditioned place aversion in mice. **(A)** The conditioned place preference (CPP) paradigm conducted in a three-chambered apparatus. During the conditioning phase mice received intraperitoneal injections of vehicle (blue) in the black chamber on odd days and CP 55,940 (red) in the white chamber on even days. **(B)** Mice injected with CP 55,940, but not vehicle, displayed a significant decrease in time spent in the drug-paired chamber. *P < 0.05 (one-way ANOVA, Welch and Brown–Forsythe test). **(C)** Representative tracks illustrating CP 55,940-induced aversion in mice.

To investigate the type of CB receptors and cellular mechanism underlying the aversive effects of CBs, we first established mouse CPP paradigm conducted in a three-chambered apparatus (Figure 1A) with CP 55,940 which is a bicyclic memetic of Δ^9^-THC (Gold et al., 1992). We found that mice preferred not to stay in the chamber paired with intraperitoneal (i.p.) injection of CP 55,940 at a high concentration of 1 mg/kg (Figure 1, vehicle-paired: pre-test 341.0 ± 18.77 S, post-test 363.1 ± 38.87 S, n = 8, *P* > 0.05; CP 55,940-paired: pre-test 310.0 ± 12.58 S, post-test 206.3 ± 29.32 S, *n* = 10, *P* < 0.05), indicative of the establishment of CPA. This is consistent with the previous reports that at relatively high concentrations, CPA is induced by the CP 55,940 in rats (McGregor et al., 1996), or by Δ^9^-THC in rats (Norris et al., 2019; Spiller et al., 2019) and mice (Ghozland et al., 2002; Cheng et al., 2004).

### The Aversive Effect of CP 55,940 Is Mediated by the CB1Rs

**FIGURE 2:**
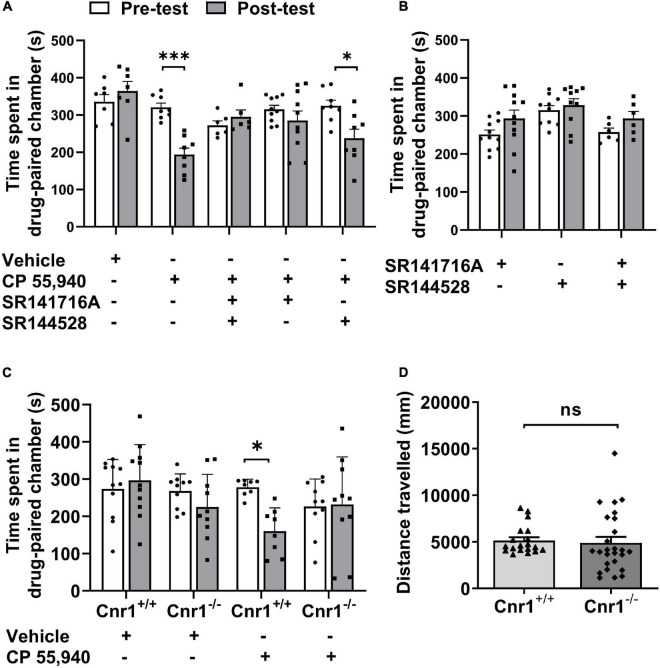
CP 55940-induced conditioned place aversion is prevented by the antagonism or systematic deletion of CB1 receptors. **(A)** Effects of antagonists of CB1R and CB2R (SR141716A and SR144528, respectively) on CP 55,940 conditioning. **(B)** Conditioning with SR141716A and SR144528 alone or in combination showed no place preference. **(C)** Effects of CB1R knockout on CP 55,940 conditioning. *P < 0.05, ^***^P < 0.001 (one-way ANOVA, Welch and Brown–Forsythe test). **(D)** Distances traveled in the conditioned place preference apparatus for *Cnr1*^+/+^ (*n* = 19) and *Cnr1*^– /–^ mice (*n* = 26). ns, not statistically significant (Student’s unpaired *t*-test).

Like Δ^9^-THC, CP 55,940 is a non-selective agonist for CB1 and CB2 receptors (Sain et al., 2009) with a high affinity (Herkenham et al., 1991; Gold et al., 1992). To analyze which CB receptor mediates CPA effect of CP 55,940, we first pharmacologically blocked actions of CB1Rs or CB2Rs. Pretreatment with selective antagonist of CB1R SR141716A, but not selective antagonist of CB2R SR144528, CPA effects of CP 55,940 were abolished (Figure 2A, CP 55,940 + SR141716A: pre-test 315.4 ± 10.80 S, post-test 285.4 ± 25.50 S, *P* > 0.05; CP 55,940 + SR144528: pre-test 324.6 ± 15.20 S, post-test 237.8 ± 24.12 S, *P* < 0.05). Conditioning with either SR141716A or SR144528 alone did not cause the bias for the drug-paired chamber (Figure 2B, SR141716A: pre-test 251.5 ± 11.52 S, post-test 293.6 ± 21.95 S, *P* > 0.05; SR144528: pre-test 315.3 ± 12.16 S, post-test 328.6 ± 16.86 S, *P* > 0.05). These data suggest that CB1Rs are responsible for the aversive effect of CP 55,940.

Then, to confirm the role of CB1Rs in the aversive effect of CP 55,940, we generate CB1R knockout (CB1R-KO, *Cnr1*^–/^*^–^*) mice with CRISPR/Cas9 system by deleting the coding DNA sequence (CDS) locating within the 2nd exon of *Cnr1* gene which encodes CB1R (Supplementary Figure 1A). The deletion of CB1Rs was verified by probe-based PCR genotyping, immunoblotting, and immunohistochemistry with specific antibody (Supplementary Figures 1B–D). Comparing with CB1R wild-type (CB1R-WT, *Cnr1*^+/+^) mice, *Cnr1*^–/^*^–^* mice had no bias for the chambers of the apparatus (Figure 2C, CB1R-WT: pre-test 273.4 ± 23.92 S, post-test 296.3 ± 29.12 S, *P* > 0.05; CB1R-KO: pre-test 268.1 ± 14.55 S, post-test 225.1 ± 27.66 S, *P* > 0.05) and displayed no locomotor deficit as the total distance traveled in the CPP apparatus was similar between the two genotypes (Figure 2D, CB1R-WT: 5,141 ± 352.4 mm; CB1R-KO: 4,893 ± 636.9 mm. *P* > 0.05). However, knockout of CB1Rs eliminated the induction of CPA by CP 55,940 (Figure 2C, CB1R-WT: pre-test 278.0 ± 7.765 S, post-test 160.2 ± 22.03 S, *P* < 0.05; CB1R-KO: pre-test 226.6 ± 23.29 S, post-test 232.1 ± 40.27 S, *P* > 0.05). Together with the results from the pharmacological antagonism against CB receptors, these sets of data indicate that the CB1Rs mediate CPA induced by CP 55,940.

### Astroglial CB1Rs Mediate the Aversive Effect of CP 55,940

**FIGURE 3:**
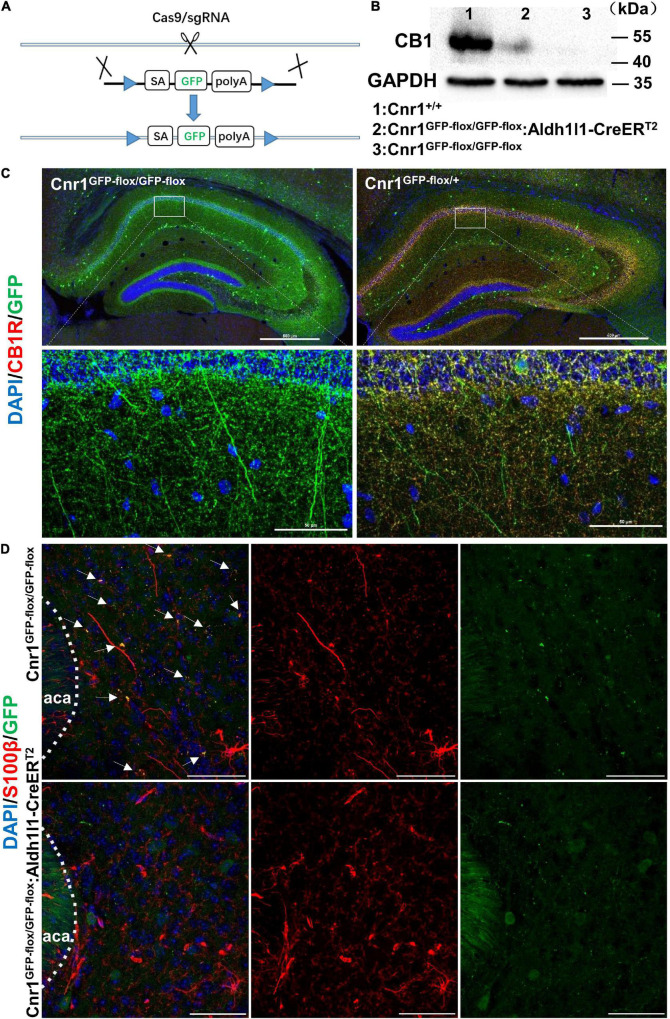
Generation and characterization of *Cnr1*^GFP– flox/GFP– flox^: *Aldh1l1*-CreER^T2^ mice. **(A)** Schematic diagram showing the generation of *Cnr1*^GFP– flox/GFP– flox^ mice by insertion of loxp-SA-GFP-pA-loxp (GFP-flox) sequence into the intron of *Cnr1* gene *via* the CRISPR/Cas9 system. **(B)** Western blotting showing the expression of CB1R in the nucleus accumbens of *Cnr1^+/+^*, *Cnr1*^GFP– flox/GFP– flox^, and *Cnr1*^GFP– flox/GFP– flox^: *Aldh1l1*-CreER^T2^ mouse brain. **(C)** Representative montage of the hippocampus showing the expression of CB1R in *Cnr1*^GFP– flox/GFP– flox^ and *Cnr1*^GFP– flox/+^ mice. Scale bar: above, 500 μm; below, 50 μm. **(D)** Representative montage of the nucleus accumbens showing the expression of GFP and S100β in *Cnr1*^GFP– flox/GFP– flox^ and *Cnr1*^GFP– flox/GFP– flox^: *Aldh1l1*-CreER^T2^ double-transgenic mice injected with tamoxifen. Arrows indicate the co-expression of GFP and S100β. Scale bar: 50 μm.

Next, to analyze whether astroglial CB receptors participate in the aversive effects of CBs, we generated transgenic mice that only express the CB1Rs in astrocytes. For this purpose, we first knocked out *Cnr1* gene by knocking in *loxp-SA-GFP-pA-loxp* (*GFP-flox*) sequence into the intron of *Cnr1* gene with CRISPR/Cas9 system, resulting in a mouse line *Cnr1*^GFP–flox/GFP–flox^ (Figure 3A). GFP reporter in this line is translated and stopped by *polyA* (*pA*), leading to the loss of CB1Rs in *Cnr1*^GFP–flox/GFP–flox^ homozygous mouse (Figures 3B,C). In contrast, there are expressions of both GFP and CB1Rs in *Cnr1*^GFP–flox/+^ heterozygous mouse (Figure 3C). Then we crossed *Cnr1*^GFP–flox/GFP–flox^ mouse with *Aldh1l1*-CreER^T2^ mouse (Hu et al., 2020; Wang et al., 2021) which specifically expresses the Cre-recombinase in astrocytes. *GFP-flox* cassette of the astrocyte in *Cnr1*^GFP–flox/GFP–flox^: *Aldh1l1*-CreER^T2^ mouse treated with tamoxifen (100 mg/kg, i.p.) is deleted by CRE-recombinase and thus CB1Rs are re-expressed only in astrocytes. Therefore, in *Cnr1*^GFP–flox/GFP–flox^: *Aldh1l1*-CreER^T2^ mouse treated with tamoxifen, GFP is deleted from astrocytes and CB1Rs are allowed to be re-expressed only in astrocytes. The establishment of *Cnr1*^GFP–flox/GFP–flox^: *Aldh1l1*-CreER^T2^ mouse line was ensured by the mild amount of re-expression of CB1Rs (Figure 3B) and dissociation of GFP with the astroglial marker S100β (Figure 3D).

**FIGURE 4:**
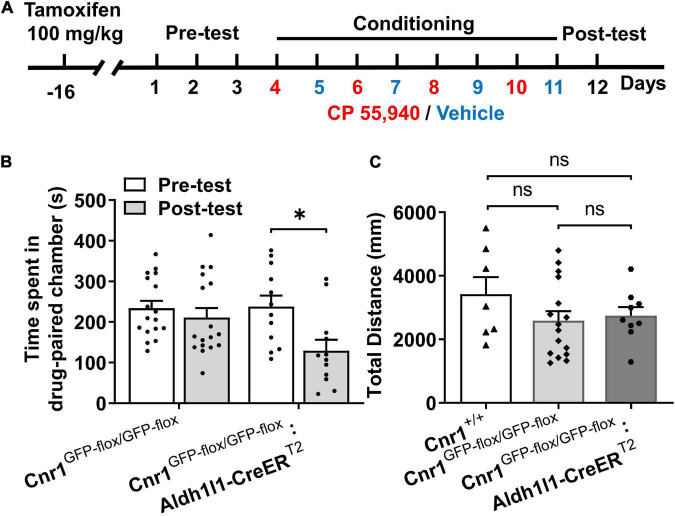
The CP 55,940 induces conditioned place aversion (CPA) in *Cnr1*^GFP– flox/GFP– flox^: *Aldh1l1*-CreER^T2^, but not CPA *Cnr1*^GFP– flox/GFP– flox^ mice treated with tamoxifen. **(A)** The conditioned place preference (CPP) procedure for conditional transgenic mice was performed 10 days after the last injection of tamoxifen (100 mg/kg, once a day for seven consecutive days). **(B)** *Cnr1*^GFP– flox/GFP– flox^:*Aldh1l1*-CreER^T2^, but not *Cnr1*^GFP– flox/GFP– flox^ mice showing conditioned place aversion effect of CP 55,940. *P < 0.05 (one-way ANOVA, Welch and Brown–Forsythe test). **(C)** Distances traveled in the CPP apparatus for *Cnr1*^+/+^, *Cnr1*^GFP– flox/GFP– flox^, and *Cnr1*^GFP– flox/GFP– flox^:*Aldh1l1*-CreER^T2^ mice. ns, not statistically significant (Student’s unpaired *t*-test).

Prior to behavioral test in the CPP paradigm, *Cnr1*^GFP–flox/GFP–flox^: *Aldh1l1*-CreER^T2^ mice were i.p. injected with tamoxifen for 7 consecutive days and remained in the homecage for 10 days to allow the reinstatement of astroglial CB1Rs (Figure 4A). Similar to CB1R knockout mice, *Cnr1*^GFP–flox/GFP–flox^ mice failed to exhibit CP 55,940-induced CPA (Figure 4B, *Cnr1*^GFP–flox/GFP–flox^: pre-test 223.8 ± 17.95 S, post-test 210.9 ± 23.60 S, *P* > 0.05). However, CPA could be induced by the CP 55,940 in *Cnr1*^GFP–flox/GFP–flox^: *Aldh1l1*-CreER^T2^ mice (Figure 4B, *Cnr1*^GFP–flox/GFP–flox^: *Aldh1l1*-CreER^T2^: pre-test 237.6 ± 27.58 S, post-test 129.4 ± 26.45 S, *P* < 0.05). There was no significant difference in the total distance traveled in the CPP apparatus among *Cnr1^+/+^*, *Cnr1*^GFP–flox/GFP–flox^, and *Cnr1*^GFP–flox/GFP–flox^: *Aldh1l1*-CreER^T2^ mice (Figure 4C, *Cnr1^+/+^* 3,419 ± 541.8 mm, *Cnr1*^GFP–flox/GFP–flox^ 2,591 ± 296.0 mm, *Cnr1*^GFP–flox/GFP–flox^: Aldh1l1-CreER^T2^ 2,746 ± 269.6 mm, *P* > 0.05), suggesting that these lines of mouse do not have locomotor deficit. Thus, re-expression of CB1Rs in astrocytes restored CPA induced by CP 55,940, indicating that astroglial CB1Rs mediate the aversive effect induced by CP 55,940.

### Astrocytes Are Activated Following Conditioned Place Aversion Induction by CP 55,940

**FIGURE 5:**
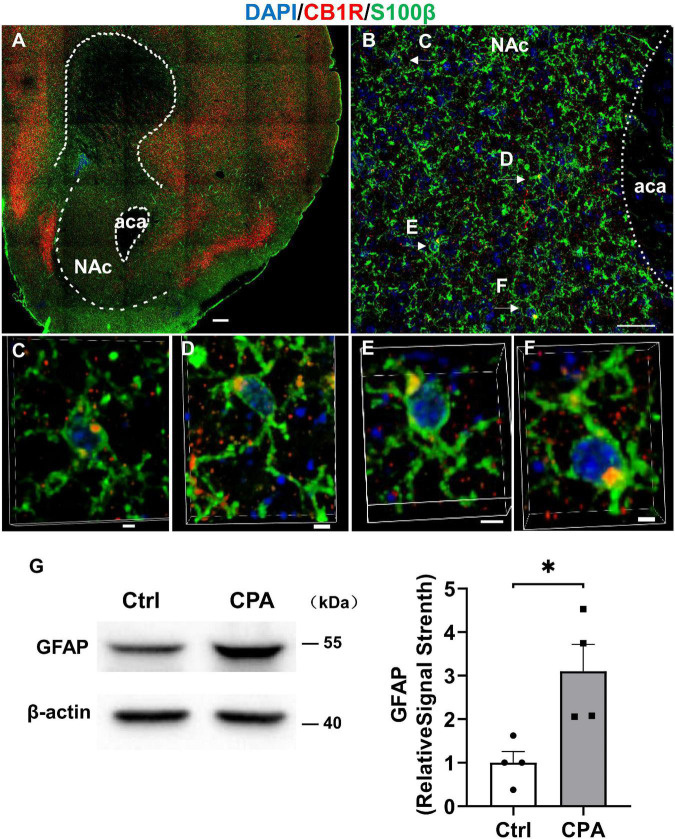
Astroglia in the nucleus accumbens express CB1R and they are activated after conditioned place aversion induction by CP 55,940. **(A)** Representative montage of the nucleus accumbens in *Cnr1*^+/+^ mouse stained for S100β (astroglial marker) and CB1R. Scale bar 250 μm. **(B)** Co-localizations of S100βand CB1R indicated by the arrows. Scale bar 25 μm. **(C–F)** 3D enlargement to illustrate the co-localizations of S100β and CB1R indicated by the arrows in panel **(B)**. Scale bar 2.5 μm. **(G)** Representative (left) and quantification of Western blotting (right) showing GFAP expression in the control mice and mice with conditioned place aversion induction. *P < 0.05 (Student’s unpaired *t*-test).

To see whether astrocytes are indeed responsive to CPA induction by CP 55,940, we detected the expression of glia fibrillary acidic protein (GFAP), another astrocytic marker, in the NAc where the expression of CB1Rs in the astrocytes was evidenced by its co-expression with S100β in our experiments (Figures 5A–F). Comparing with the control of vehicle conditioning, the CPA induction by CP 55,940 conditioning significantly increased GFAP expression in the NAc (Figure 5G, *P* < 0.05), indicating that astrocytes are activated following CPA induction.

### Inhibition of COX-2 Eliminates the Aversive Effect of CP 55,940

**FIGURE 6:**
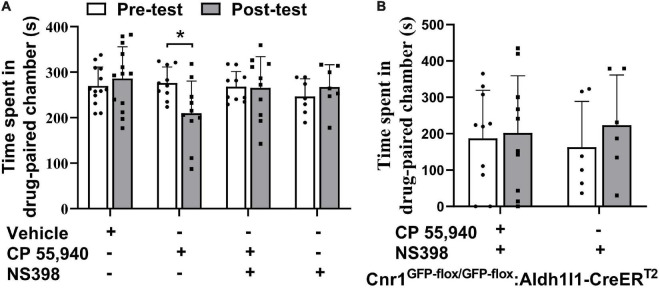
Inhibition of cyclooxygenase-2 blocks conditioned place aversion induction by CP 55,940. **(A)** Statistical data showing that NS398 (specific cyclooxygenase-2 inhibitor, 10 mg/kg) does not induce conditional place preference, while it prevents conditioned place aversion (CPA) induction by CP 55,940 in *Cnr1*^+/+^ mice. NS398 is intraperitoneal injected 30 min before CP 55,940. **(B)** Statistical data showing that NS398 does not induce conditional place preference, while it prevents CPA induction by CP 55,940 in *Cnr1*^GFP– flox/GFP– flox^: *Aldh1l1*-CreER^T2^ mice treated with tamoxifen. *P < 0.05 (one-way ANOVA).

The previous study illustrated that high doses of Δ^9^-THC, e.g., 5 mg/kg and 10 mg/kg, can cause synaptic and memory impairments through COX-2 signaling and astroglial CB1Rs (Han et al., 2012; Chen et al., 2013). Since we have observed that astroglial CB1Rs were activated by CP 55,940 and mediated CPA effect of CP 55,940 at a dose of 1 mg/kg, we proposed that the aversive effect of CP 55,940 is mediated through astrocytic COX-2 signaling. Therefore, we tested whether inhibition of COX-2 is able to eliminate the CPA effect of CP 55,940. We found that both *Cnr1*^+/+^ (Figure 6A, CP 55,940 + NS398: pre-test 268.1 ± 10.61 S, post-test 265.6 ± 21.74 S, *P* > 0.05) and *Cnr1*^GFP–flox/GFP–flox^: *Aldh1l1*-CreER^T2^ (Figure 6B, CP 55,940 + NS398: pre-test 187.3 ± 41.73 S, post-test 201.7 ± 49.93 S, *P* > 0.05) mice did not exhibit CPA when specific COX-2 inhibitor, NS398 (10 mg/kg i.p. injection), was administrated 30 min before CP 55,940 conditioning. These data demonstrate that aversive effect of CP 55,940 is mediated through astrocytic COX-2 signaling.

## Discussion

In this study, we first found CP 55,940 at a concentration of 1 mg/kg induces CPA in mouse. Next, we found that the induction of CPA by CP 55,940 is prohibited by the selective inhibitor of CB1Rs or systematic knockout of *cnr1* gene encoding CB1Rs. Then, by taking advantage of *Cnr1*^GFP–flox/GFP–flox^: Aldh1l1-CreER^T2^ mice whose CB1Rs are knocked out by inserting *loxp-SA-GFP-pA-loxp* sequence into the intron of *Cnr1* gene and can be reinstated only in the astrocytes by the treatment with tamoxifen, we found that CPA effect of CP 55,940 is mediated by the astroglial CB1Rs through COX-2 signaling. Taken together, these findings provide novel understanding of cellular and molecular mechanisms underlying direct aversive effect of CBs.

Cannabinoids include phytocannabinoids, synthetic CBs, and endogenous CBs. As phytocannabinoids and synthetic CBs are not endogenously produced by animals, CBs can also be grouped into two classes: exogenous and endogenous CBs. Both classes of CBs have been documented bivalent effects on the reward and aversion processing of themselves or non-CB psychoactive substances. At relatively lower concentrations, they produce rewarding effects, while at higher concentrations they produce aversive effects (Scherma et al., 2008; Murray and Bevins, 2010; Norris et al., 2019). The rewarding effects of CBs are considered to be mediated by CB1Rs through the interaction with mesolimbic dopaminergic (Centonze et al., 2004; Cheer et al., 2007; Oleson and Cheer, 2012; Navabpour et al., 2021) and opioid systems (Braida et al., 2001; Ghozland et al., 2002; Norris et al., 2019). However, it is controversial regarding the types of CB receptors mediating the aversive effect of CBs. Within the CeA and VTA, local application of selective CB1Rs agonist arachidonylcyclopropylamide leads to rewarding reinforcement, while within the mPFC it leads to aversive behaviors (Navabpour et al., 2021). Systematic administration of high doses of non-specific agonist Δ^9^-THC or WIN55,212-2 produces suppressive effects on the brain-stimulation reward that can be blocked by CB2R antagonist (Spiller et al., 2019). Using transgenic knockout mice, Li et al. (2021) found that CB1R knockout attenuates Δ^9^-THC-induced CPP at a low dose and enables a high dose of Δ^9^-THC to induce CPA, and that CB2 receptor knockout display robust CPP, but no CPA (Li et al., 2021).

The controversy may arise due to several factors in the experimental designs of different studies, such as experimental models for the detection of aversion effects, brain regions, agonists or antagonists of CB receptors, and lines and genetics of animals. Some studies detected indirect effects of CBs on aversion, e.g., effects of CBs on the brain-stimulation reward of rats (Spiller et al., 2019), cocaine or ethanol reinforcing of mice (Aracil-Fernandez et al., 2012; Ortega-Alvaro et al., 2015), aversive memory of mice (Soria-Gomez et al., 2015), aversive responses in the rat model of panic attacks (Viana et al., 2019). Some other studies detected the direct effect of aversion against CBs in rat (McGregor et al., 1996; Scherma et al., 2008) or mice (Han et al., 2017; Li et al., 2021). In our present study, we showed the potent CB receptor agonist CP 55,940, an synthetic analog of Δ^9^-THC, is able to induce CPA directly at a high dose in mice, consistent with previous reports from rats (McGregor et al., 1996). CP 55,940 is failed to induce CPA when CB1R, but not CB2R, was blocked by specific antagonist, suggesting that CB1R mediates aversive effect of CP 55,940. This suggestion was substantiated by the experiments with CB1R knockout mice (Figures 2, 4) showing no CP 55,940-induced CPA. Therefore, our results are consistent with the previous findings that support a role CB1R (Scherma et al., 2008; Soria-Gomez et al., 2015; Han et al., 2017; Navabpour et al., 2021), but contradictory to other findings that support a role CB2R (Aracil-Fernandez et al., 2012; Ortega-Alvaro et al., 2015; Spiller et al., 2019; Li et al., 2021), in CB-associated modulation of aversive effects. Although CP 55,940 is an analog of Δ^9^-THC, it differs from Δ^9^-THC not only in the efficacy of activating CB receptors (McGregor et al., 1996; Singh et al., 2011), but also possibly in the receptor mechanisms of regulating brain functions which include temperature (Grim et al., 2016) as well as rewarding and aversion responses (Singh et al., 2011). In our hands, 1 mg/kg CP 55,940 produces CPA, while Li et al. (2021) reported the CPP of 1 mg/kg Δ^9^-THC in mice. With regard to the direct effect of CBs on mice aversive behavior, our data convincingly reveal a role CB1R in mediating the CPA effect of CP 55,940 at a concentration of 1 mg/kg.

The CB1Rs and CB2Rs are presented in both neuronal and glial cells widely across the brain. Recently, conditional-targeted knockout of CB1Rs has been helping to analyze the CB actions on rewarding and aversion processing through particular cell subpopulations. Until now, this strategy has revealed that CB1Rs in the GABAergic medial spiny neurons contribute to locomotor sensitization and striatal activation of ERK pathway of mice in response to cocaine or D-amphetamine (Corbille et al., 2007), and that dopaminergic CB2Rs influence “tetra effects” of CBs and mediate CPP effect of alcohol (Liu et al., 2020). Meanwhile, CB1Rs on the glutamatergic neurons have been determined to be responsible for Δ^9^-THC-induced CPA effect (Han et al., 2017). It has not been investigated whether astroglial CB receptor participates in rewarding or aversive effects of addictive substances. In this study, we demonstrated that the prevention of the induction of CPA effect of CP 55,940 by pharmacological inhibition or systematic deletion of CB1Rs is rescued by conditional reinstatement of CB1Rs in the astrocytes. Therefore, neuronal and astrocytic CB1Rs may differentially or coordinately participate in CPA effects of some CBs. A combined use of both cell type-specific conditional knockout and cell type-specific conditional expression of CB1Rs should be designed in the future experiments to precisely determine the contributions of different types of cells in aversive effects of CBs.

Furthermore, we demonstrated that astrocytes are activated by CP 55,940 conditioning and specific inhibition of COX-2 prevents the induction of CPA effect of CP 55,940 in mice with wild type CB1Rs or conditional expression of CB1Rs only in astrocytes. COX-2 is an inducible enzyme playing an important role in inflammation of many central and peripheral diseases (Chen, 2010). Our previous research showed that hippocampal COX-2 production after Δ^9^-THC treatment is mainly from astrocytes and Δ^9^-THC induces synaptic and memory impairments via COX-2 signaling causing loss of neuronal excitation/inhibition balance (Chen et al., 2013). In addition to astrocytes, microglia play roles in COX-2-mediated neuroinflammation (Font-Nieves et al., 2012) and astrocyte-microglial interactions (Bernaus et al., 2020; Matejuk and Ransohoff, 2020), they may be activated during CPA procedure and contribute to CPA formation. Thus, CP 55,940-induced CPA effect could be just one of the behavioral phenotypes of COX-2-mediated neuroinflammation causing a wide range of neuronal damages. Our data suggest that selective inhibition of COX-2 by NSAIDs or cannabidiolic acid which is the acidic precursor of cannabidiol, a non-psychoactive component of cannabis (Takeda et al., 2008), may be useful for treating not only cognitive impairments (Chen et al., 2013), but also the aversive effects and possibly some other side effects of medical or recreational administrations of marijuana.

## Conclusion

Astroglial COX-2 production by exogenous CBs may commonly lead to many detrimental and adverse outcomes on neuronal functions, while selective COX-2 inhibition or precise isolation of astroglial CB1 receptors activity may be useful for treating aversion and other unwanted reactions of medical and recreational applications of marijuana.

## Materials and Methods

### Animals

The experimental C57BL/6J mice were purchased from Guangdong Medical Laboratory Animal Center. Animals were kept in a specific pathogen free room with controlled temperature (22–24^°^C), humidity (50–75%), light conditions (12 h/12 h light-dark cycle, 8:00–20:00), and free access to food and water. All the mice used in this study were male and at age of 8 weeks.

*Cnr1*^–/–^ mice were produced in a C57BL/6J background mice by Beijing Vitalstar Biotechnology Co., Ltd. Briefly, *Cnr1*^–/–^ mice were generated via a CRISPR/Cas9 system. The specific guide RNAs were designed (gRNA1: 5′-GGGTAGTTAGGCTTCAGATT-3′; gRNA2: 5′-GGGATCCCAAGGTTAACATG-3′) to delete the CDS within the 2nd exon of *Cnr1* gene. The targeting constructs include the Cas9 mRNA and guide RNAs were injected into C57BL/6J zygotes. Then these zygotes were selected and transplanted into pseudopregnant mice to generate the founder mice. The mice were screened using PCR analysis with specific primers as follows: forward primer (F, 5′-TCATGTTAGCCATCTGCATTTCCA-3′) and reverse primer (R, 5′-TCACCCTGCTACATCACCACTCCT-3′) or wild-type reverse primer (WT-R, 5′-GATGTCTTTGAACC AAAGTCAATGGTC-3′). The PCR was programmed carried out with initial heating to 95^°^C for 1.5 min, then 35 cycles of 95^°^C for 25 s, 58.5^°^C for 30 s, and 72^°^C for 30 s, with a final extension at 72^°^C for 1.5 min. The size of the PCR products was used for distinguishing the genotype of the mice: wild-type (F + R, no product; F + WT-R, 680 bp), homozygote (F + R, 420 bp; F + WT-R, no product).

*Cnr1*^GFP–flox/GFP–flox^ mice were produced in a C57BL/6J background mice by Beijing Vitalstar Biotechnology Co., Ltd. Briefly, *Cnr1*^GFP–flox/GFP–flox^ mice were generated *via* a CRISPR/Cas9 system using Cas9 mRNA, single guide RNA (GGGAAAATCAGACCTCGTGGTGG) and a donor, which were co-injected into C57BL/6J zygotes. Then these zygotes were transplanted into pseudopregnant mice. The sgRNA-directed Cas9 endonuclease cleavage was occurred near the intron and created the double-stranded breaks (DSBs). The DSBs stimulated DNA repair through homologous recombination and insertion of *loxp-SA-GFP-pA-loxp* sequence into the intron of *Cnr1* gene ultimately. The mice were screened using PCR analysis with specific primers as follows: forward primer *cnr1*-seq-F (5′-GCGTGCTCATTGTTAAGAAGACACA-3′) and reverse primer *cnr1*-eGFP-R (5′-GCTCCTCGCCCTTGCTCACCAT-3′) or *cnr1*-seq-R (5′-TCTCACAGGCAAGCCAGAAAGG-3′). The PCR was programmed carried out with initial heating to 94^°^C for 4 min, then 38 cycles of 94^°^C for 20 s, 62^°^C for 25 s, and 72^°^C for 25 s, with a final extension at 72^°^C for 2 min. The size of the PCR products was used for distinguishing the genotype of the mice: wild-type (*cnr1*-seq-F + *cnr1*-eGFP-R, no product; *cnr1*-seq-F + *cnr1*-seq-R, 260 bp), homozygote (*cnr1*-seq-F + *cnr1*-eGFP-R, 390 bp; *cnr1*-seq-F + *cnr1*-seq-R, no product).

*Aldh1l1*-CreER^T2^ transgenic mice were generated and characterized previously (Hu et al., 2020; Wang et al., 2021). *Aldh1l1*-CreER^T2^ mice were crossed with *Cnr1*^GFP–flox/GFP–flox^ mice to generate *Cnr1*^GFP–flox/GFP–flox^: *Aldh1l1*-CreER^T2^ mice.

### Conditioned Place Preference Procedure

The full CPP procedure is illustrated in Figure 1A, which consisted of three phases such as pre-test, conditioning, and post-test. The CPP apparatus (GZfeidi, Guangzhou, China) consists of two large chambers (720 mm × 250 mm × 320 mm) plus a smaller middle chamber (110 mm × 250mm × 320 mm). The left chamber is black with a hole floor, and the right chamber is white with a grid floor. The middle chamber has two doors that can be opened, allowing the mice to move freely. There is a camera on the top of each chamber. During the pre-test phases (days 1–3), the mice were placed into the middle chamber and allowed to explore the three chambers for 15 min. Any mice that showed an initial unconditioned preference for the white chamber were removed from the experiment. As a result, only the animals that preferred the black chamber were included in the next phase. On conditioning day 1, each mouse received the i.p. injection of the drug and then was placed into the white chamber for 30 min. On conditioning day 2, each mouse was injected with the vehicle and then was placed into the black compartment for 30 min. This procedure was repeated for four times. On the test day (post-test), neither drug nor vehicle was injected. The test was run under the identical parameters to the pre-test sessions. The time spent in each compartment was measured as an indicator of reinforcing properties.

### Immunohistochemistry

Mice were anesthetized with i.p. injection of 75% pentobarbital sodium (Sigma #P3761), and then cardiac perfusion was performed with 4^°^C saline (0.9% NaCl), followed by 4% paraformaldehyde (PFA) in phosphate-buffered saline (PBS). The tissues were placed in 4% PFA at 4^°^C for 24 h, and then dehydrated with 30% sucrose in PBS at 4^°^C for 72 h. Tissues were embedded with OCT (Tissue-Tek #4583) and cryo-sectioned (Leica #CM1850-1-1) at 30 μm for immunohistochemical staining. The brain slices were washed three times with PBS for 15 min and incubated with PBS containing 5% BSA and 1% Triton X-100 at room temperature for 1.5 h, and then incubated with CB1 receptor (C-Term) polyclonal antibody (1:5,000, Cayman #10006590), anti-S100 monoclonal antibody (1:200, Abcam #ab4066) or recombinant anti-S100 beta monoclonal antibody (1:200, Abcam #ab52642) overnight at 4^°^C. After washing, slices were incubated with Alexa Fluor goat anti-rabbit 488 (ZSGB-Bio #ZF-0511) or 594 (ZSGB-Bio #ZF-0516) or goat anti-mouse 488 (ZSGB-Bio #ZF-0512) or 594 (ZSGB-Bio #ZF-0513) at room temperature for 1.5 h, and then stained with DAPI (1 μg/ml, Sigma #D9542), and finally washed three times with PBS for 15 min. The brain slices were placed on coverslips and inspected by Nikon A1R confocal microscope.

### Immunoblotting

Cells and tissues were homogenated in RIPA lysis buffer containing PMSF and were fractionated on 10% SDS-polyacrylamide gels, and transferred to polyvinylidene difluoride (PVDF, Millipore) membranes. The membrane was incubated with 5% defatted milk for 1 h at room temperature, and then incubated overnight with the primary antibody at 4^°^C. The membranes were washed with 0.1% Tween 20 in TBS for three times and incubated with horseradish peroxidase-conjugated secondary antibodies in TBST for 1h at room temperature. Chemiluminescence Apparatus (Bio-Rad) was used to detect the desired signals. Analysis was quantified by ipwin32 software. The following primary antibodies were used: anti-glial fibrillary acidic protein monoclonal antibody (1:1,000, Sigma #MAB360); anti-COX-2 (mouse) polyclonal antibody (1:200, Cayman #aa 570-598); anti-β-actin monoclonal antibody (1:4,000, Sigma #A2228); anti-GAPDH monoclonal antibody (1:5,000, Proteintech #60004-1-Ig).

### Drugs

CP 55,940 (Sigma #C1112), SR141716A (Tocris #0923), SR144528 (Cayman #9000491), and NS398 (Abcam #ab120295) were dissolved in a vehicle consisting of 10% DMSO (Sigma #V900090), 10% Tween 80 (Sigma #P4780) and 80% saline. Tamoxifen (Sigma #T5648) was dissolved in 10% ethanol (Sigma #32205), and 90% corn oil (Sigma #C8267).

### Statistical Analysis

Data are presented as mean ± S.E.M. The statistical difference was analyzed by Student’s *t*-test or one-way ANOVA using SPSS 22.0 and the statistical graphs were generated with GraphPad Prism 8.0 software. Statistical significance is defined as *P* < 0.05.

## Data Availability Statement

The raw data supporting the conclusions of this article will be made available by the authors, without undue reservation.

## Ethics Statement

The animal study was reviewed and approved by the Southern Medical University Animal Ethics Committee.

## Author Contributions

YG, T-MG, and RC contributed to the experimental design. WMZ, YG, T-MG, and RC established the methodology. JC, KL, WJZ, and ZL performed the experiments. JC analyzed the data. ZG, XT, JWZ, JPZ, and SL helped to perform the experiments and maintain the animals. RC and JC wrote the manuscript. All authors contributed to the finalization and approved the content of the manuscript.

## Conflict of Interest

The authors declare that the research was conducted in the absence of any commercial or financial relationships that could be construed as a potential conflict of interest.

## Publisher’s Note

All claims expressed in this article are solely those of the authors and do not necessarily represent those of their affiliated organizations, or those of the publisher, the editors and the reviewers. Any product that may be evaluated in this article, or claim that may be made by its manufacturer, is not guaranteed or endorsed by the publisher.

## Supplementary Material

Supplementary Figure 1 Generation and characterization of CB1R knockout (*Cnr1*^–/^*^–^*) mice. **(A)** Schematic diagram showing the generation of *Cnr1*^–/^*^–^* mice by deleting coding sequence (CDS) within the 2nd exon via the CRISPR/Cas9 system. **(B)** Representative PCR genotyping for *Cnr1* knockout mice with forward (For.), reverse (Rev.) and wild type reverse (WT-Rev.) primers. **(C)** Western blotting showing the expression of CB1R was missing in *Cnr1*^–/^*^–^* mice. **(D)** Representative Immunohistochemical montage of the cortex and hippocampus of *Cnr1*^–/^*^–^* and *Cnr1*^+/+^ mice mouse showing the expression of CB1R was missing in *Cnr1*^–/^*^–^* mice. Scale bar: 100 μm.
