# Supplementary figures and images for: Astroglial CB1 Cannabinoid Receptors Mediate CP 55,940-Induced Conditioned Place Aversion Through Cyclooxygenase-2 Signaling in Mice
Source: Front Cell Neurosci. 2021 Nov 23;15:772549. doi: 10.3389/fncel.2021.772549 (PMC8650095; doi:10.3389/fncel.2021.772549)

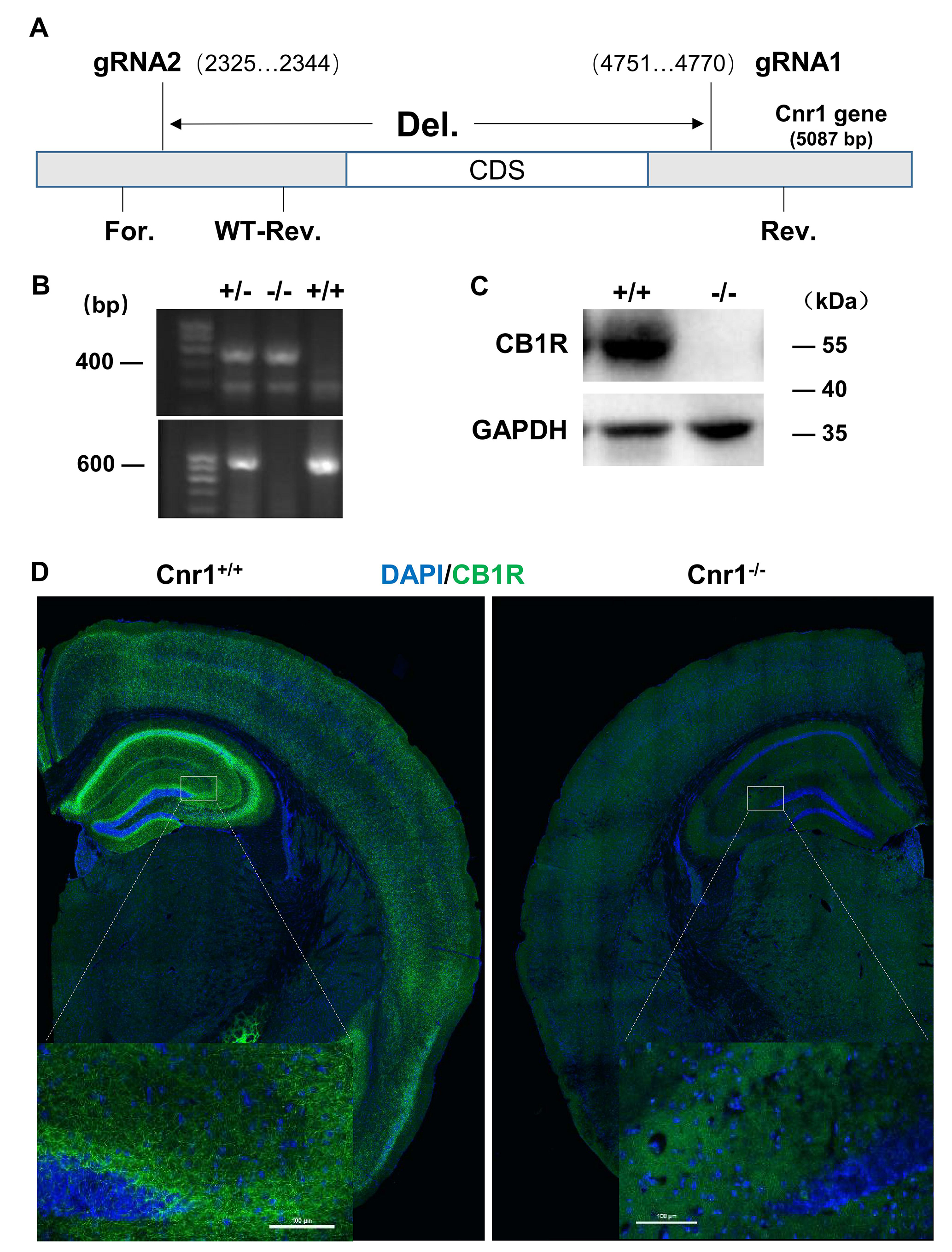

Supplement: Supplementary Figure 1 — Generation and characterization of CB1R knockout (Cnr1–/–) mice. (A) Schematic diagram showing the generation of Cnr1–/– mice by deleting coding sequence (CDS) within the 2nd exon via the CRISPR/Cas9 system. (B) Representative PCR genotyping for Cnr1 knockout mice with forward (For.), reverse (Rev.) and wild type reverse (WT-Rev.) primers. (C) Western blotting showing the expression of CB1R was missing in Cnr1–/– mice. (D) Representative Immunohistochemical montage of the cortex and hippocampus of Cnr1–/– and Cnr1+/+ mice mouse showing the expression of CB1R was missing in Cnr1–/– mice. Scale bar: 100 μm. [file Image_1.JPEG]
