# Supplementary material for: Astroglial CB1 Cannabinoid Receptors Mediate CP 55,940-Induced Conditioned Place Aversion Through Cyclooxygenase-2 Signaling in Mice
Source: Front Cell Neurosci. 2021 Nov 23;15:772549. doi: 10.3389/fncel.2021.772549 (PMC8650095; doi:10.3389/fncel.2021.772549)

Source data of fIg 3B - original gel of CB1R

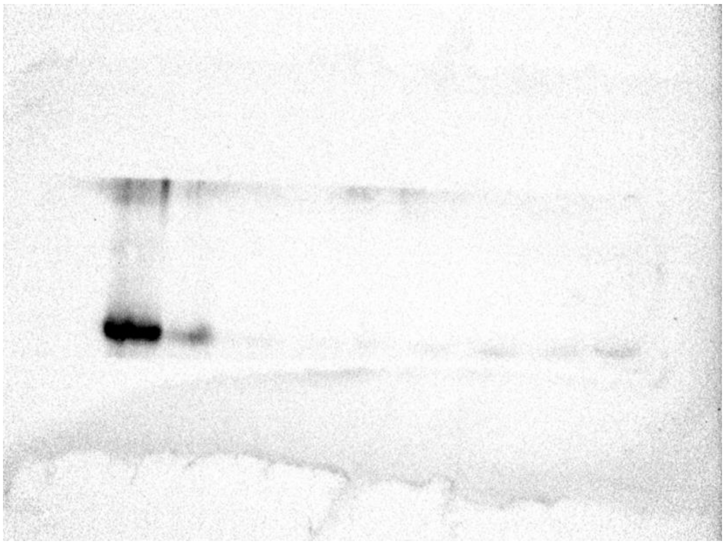

Source data of fIg 3B - original gel of GAPDH

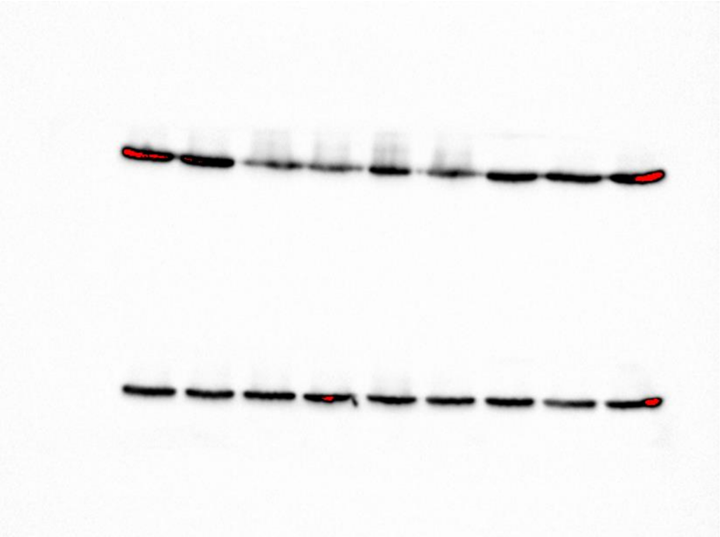

Source data of Fig 3C - original image of heterozygote 60x-1001-maxip

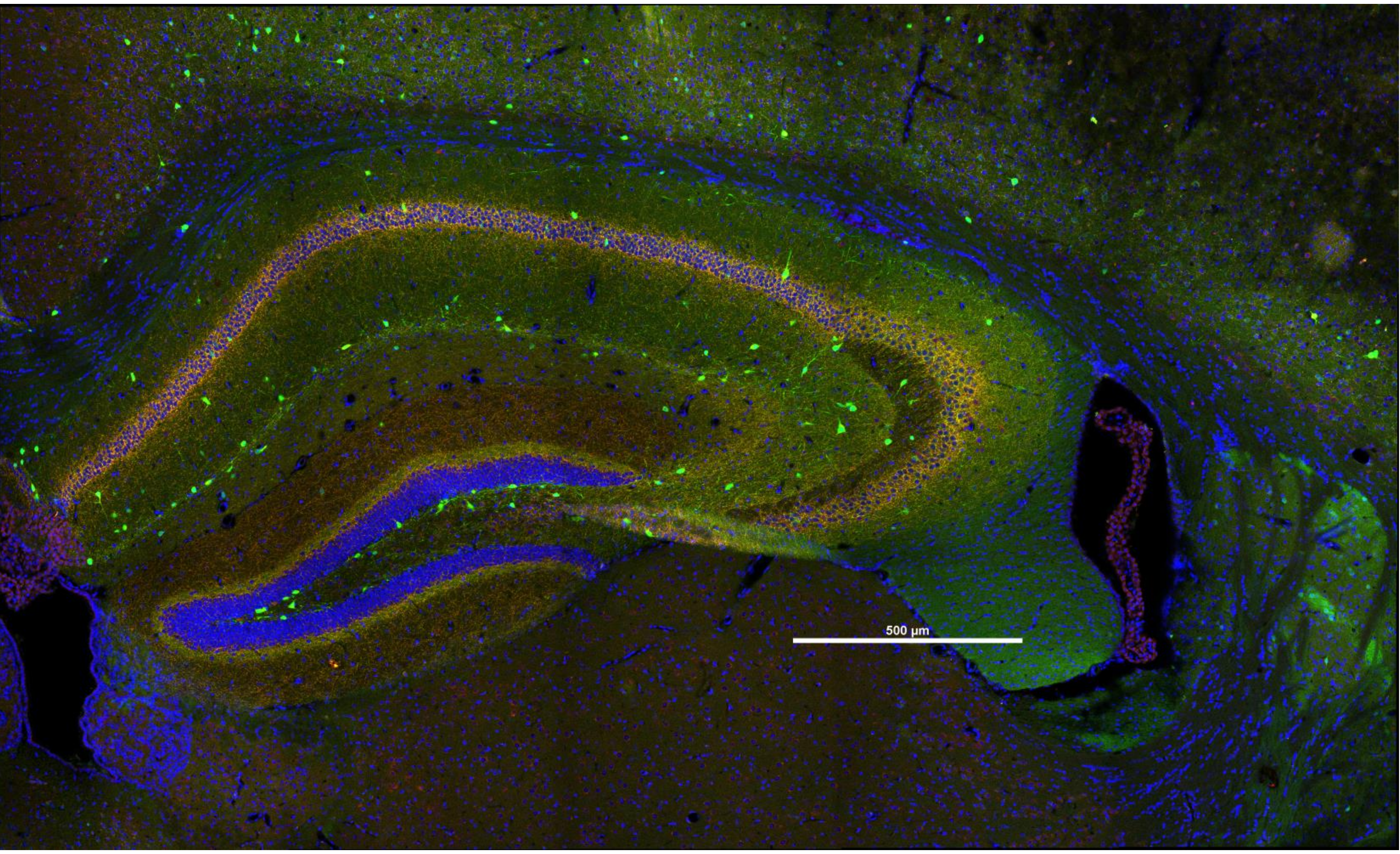

Source data of Fig 3C - original image of heterozygote 20x-r

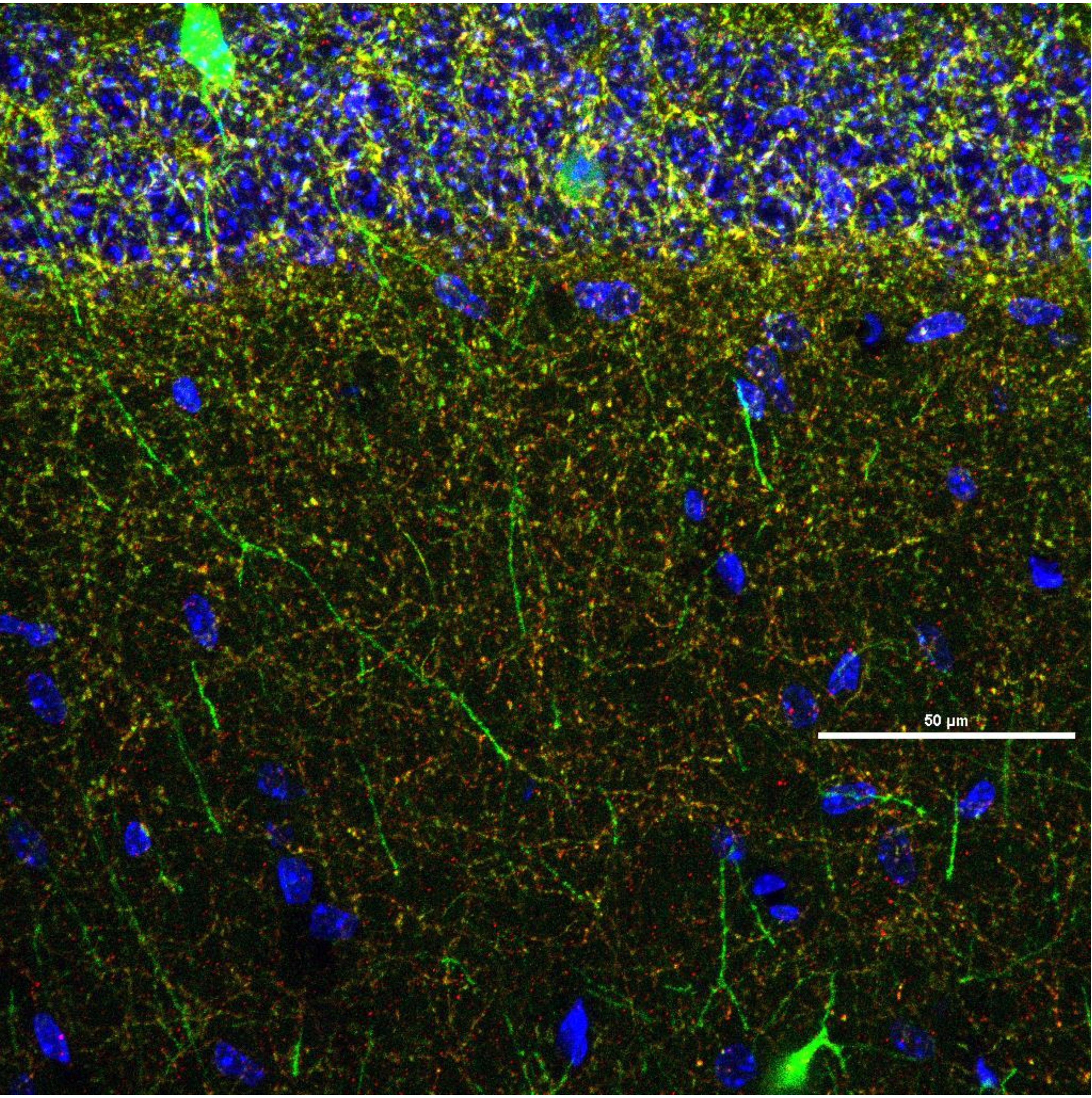

Source data of Fig 3C - original image of homozygote 20x-r

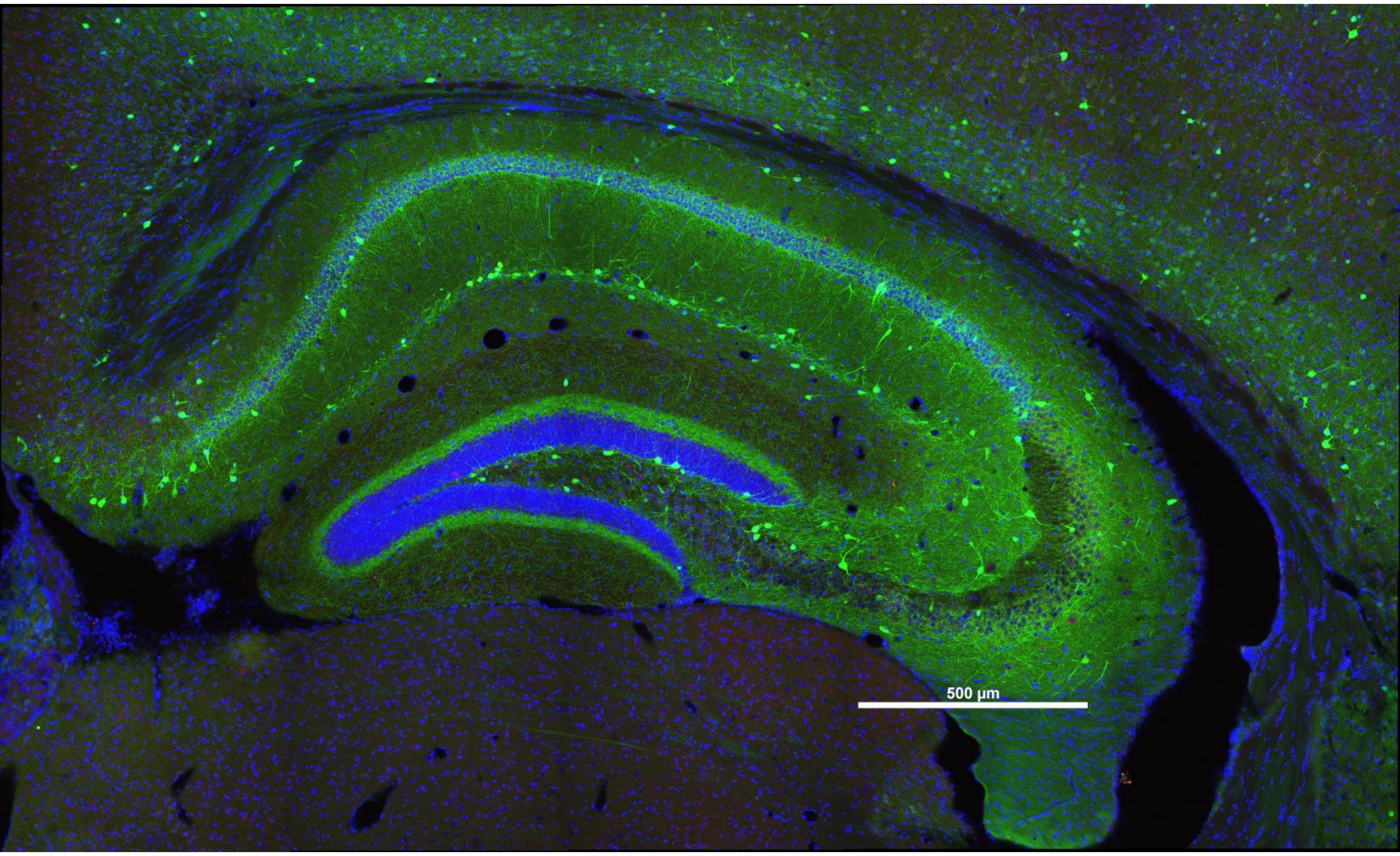

Source data of Fig 3C - original image of homozygote 20x-r

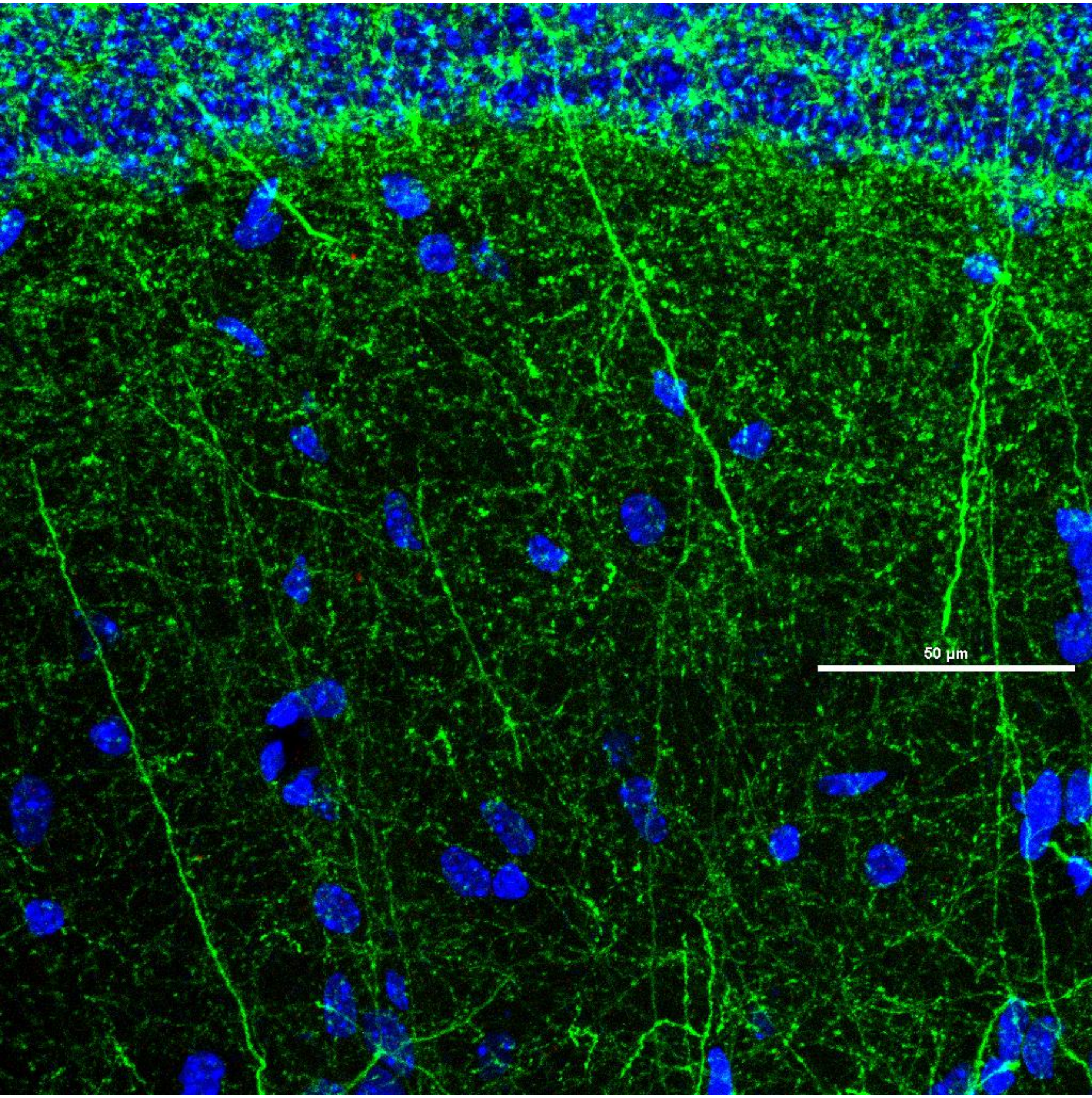

Source data of Fig 3D - original image ($Cnr1^{GFP\text{-}floxGFP\text{-}flox}$)
G60x-1001-MaxIP_RGB

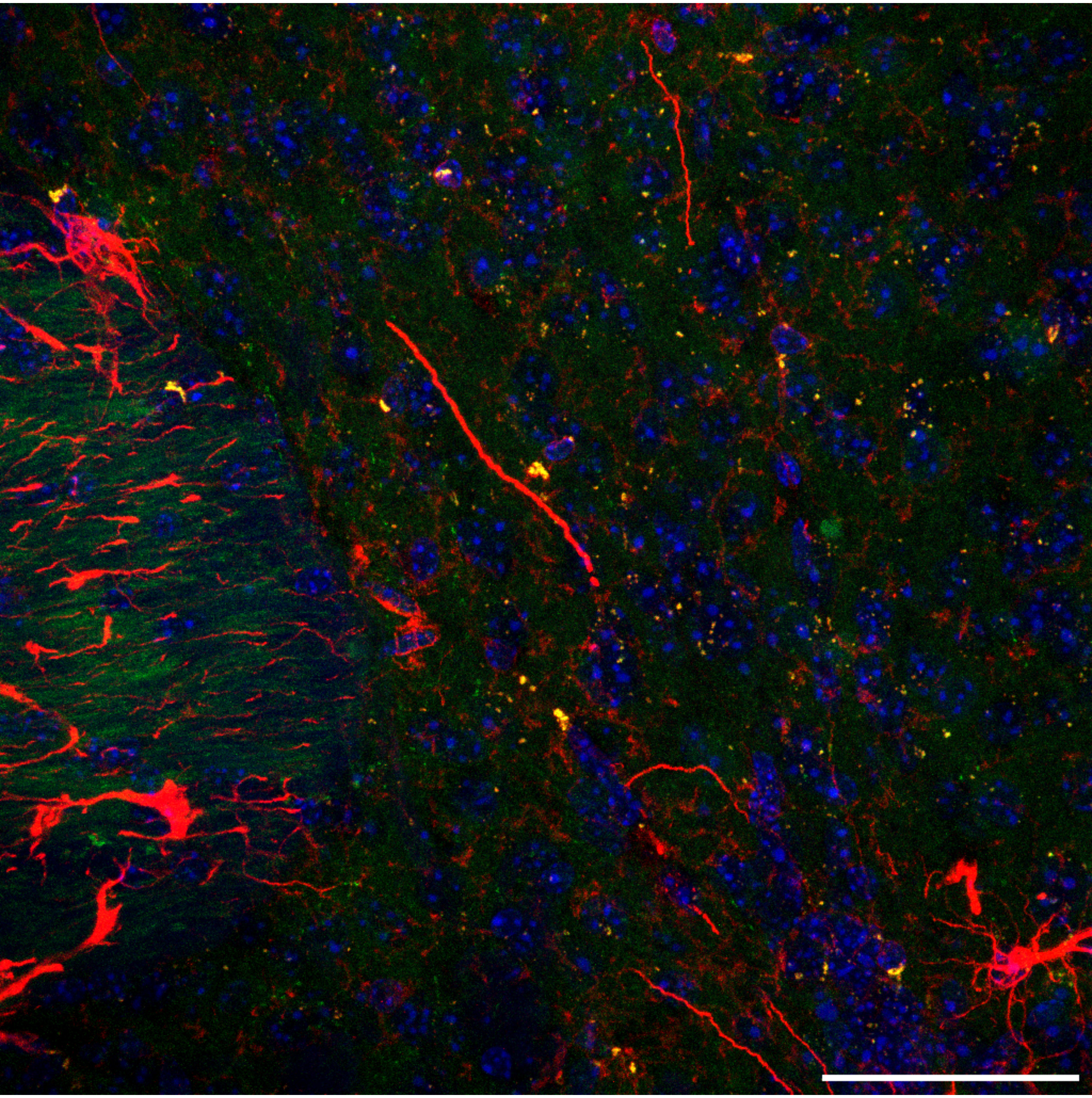

Source data of Fig 3D – original image (Cnr1$^{GFP-floxGFP-flox}$:Aldh1l1-CreER^{T2}) GA60x-1003-MaxIP_RGB

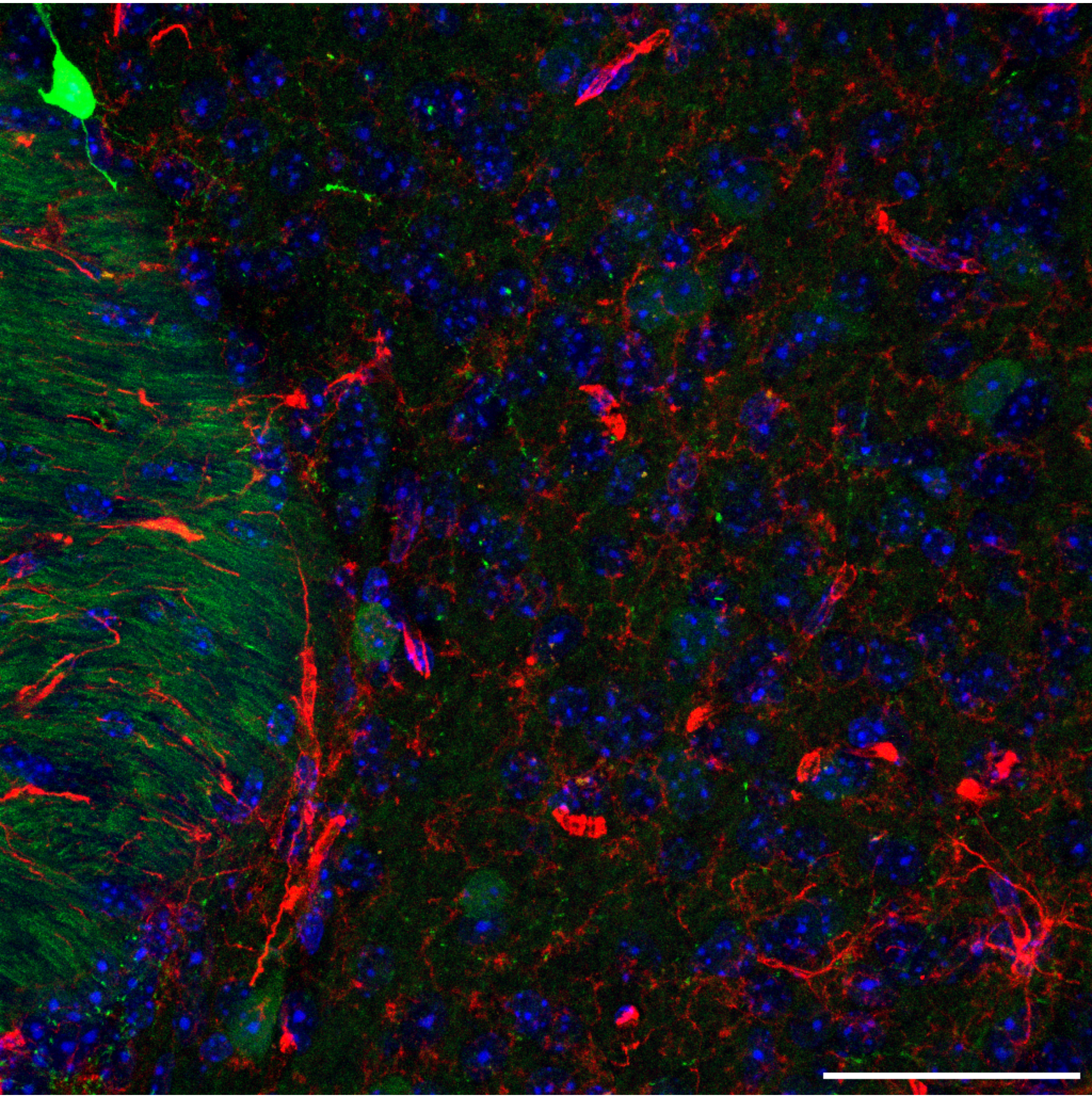

Source data of Fig 5A – original image 20x

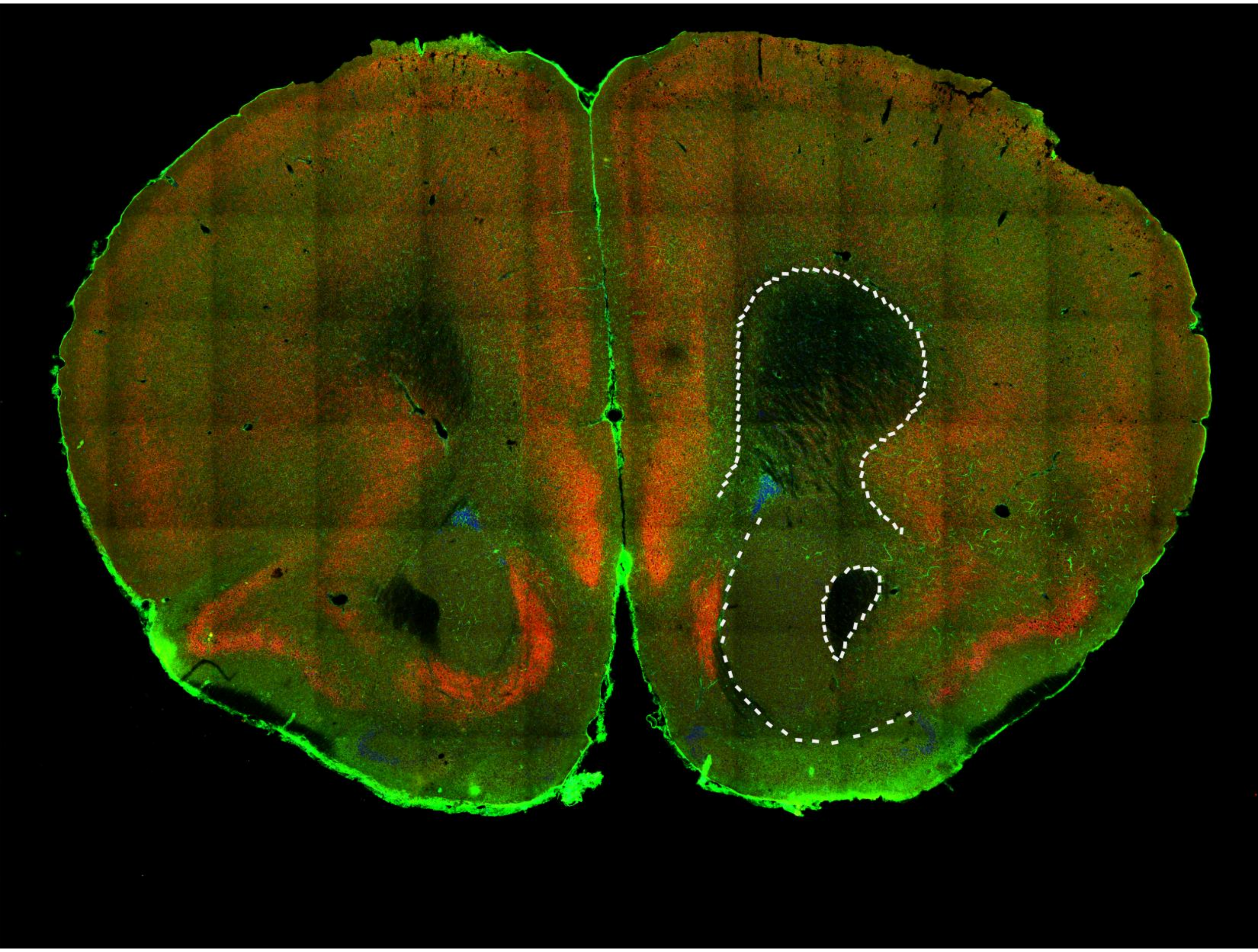

Source data of Fig 5B – original image 60x

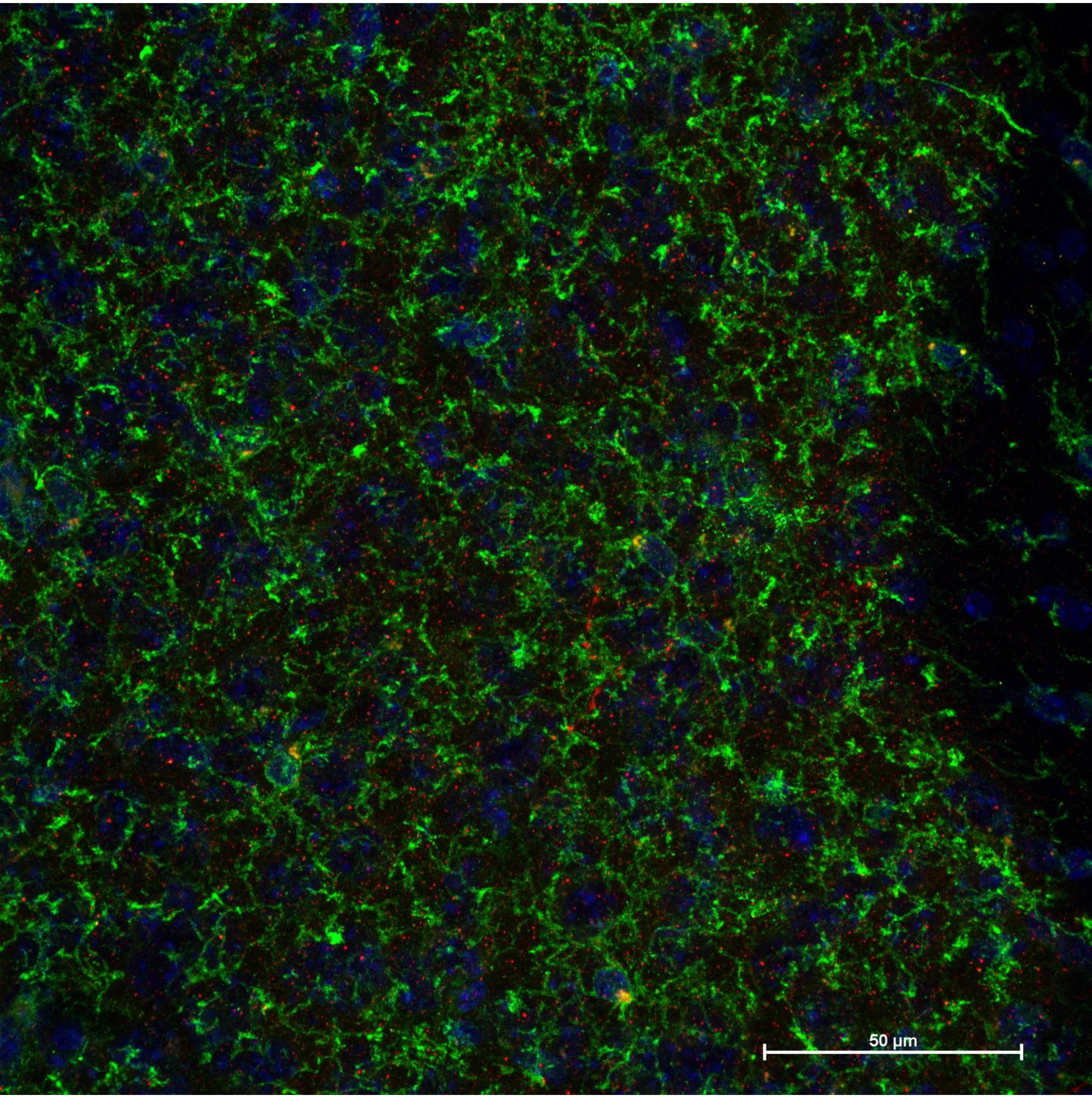

Source data of Fig 5G – original gel of GFAP

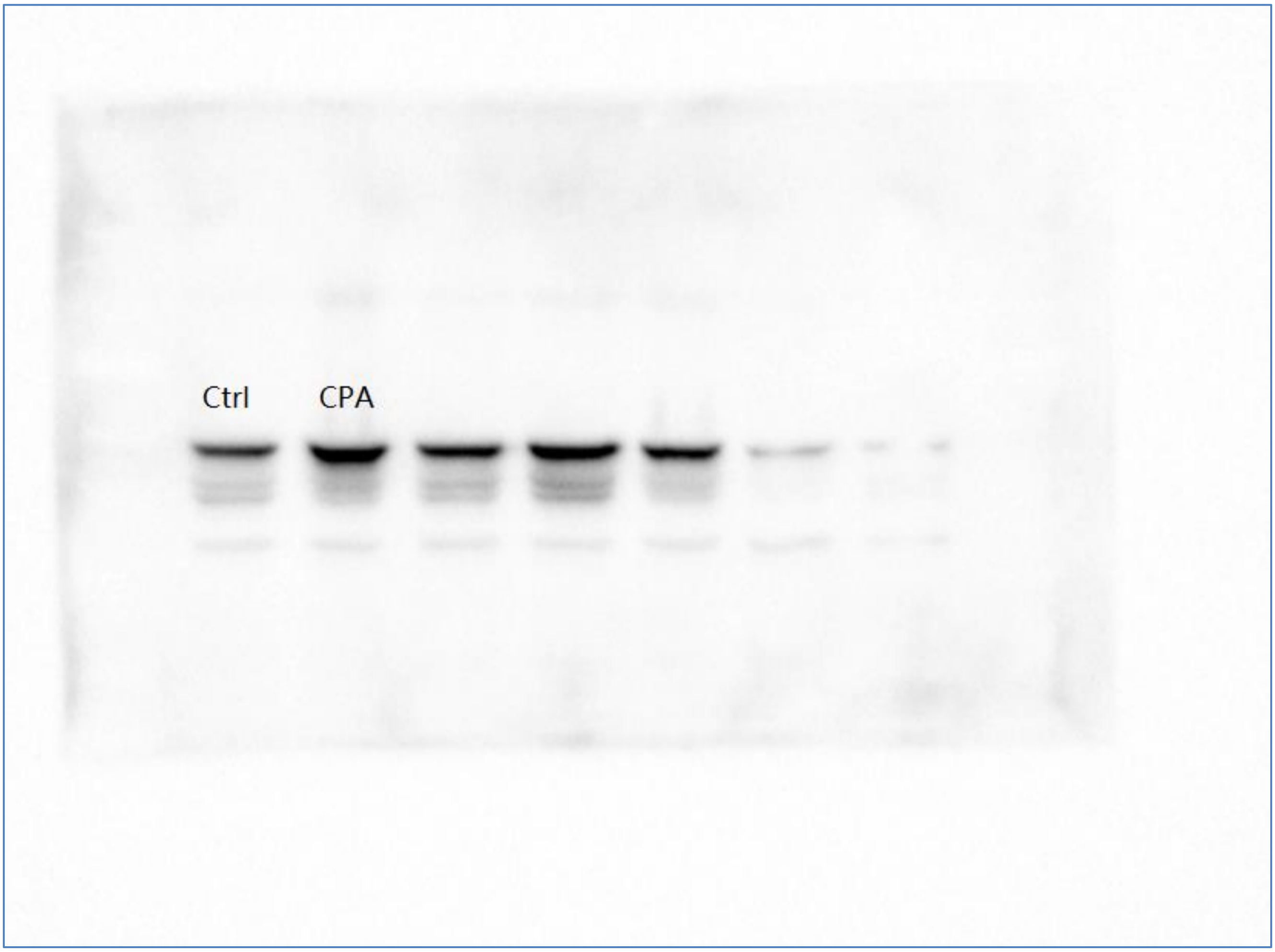

Source data of Fig 5G – original gel of actin

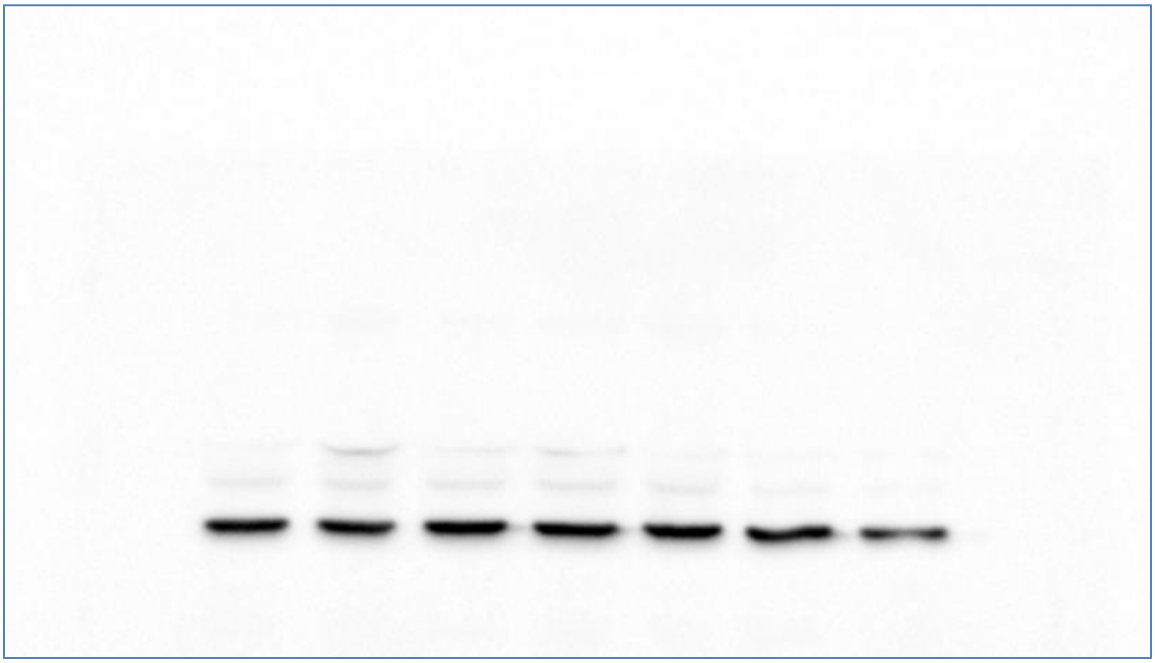

Source data of Suppl Fig 1B – original PCR

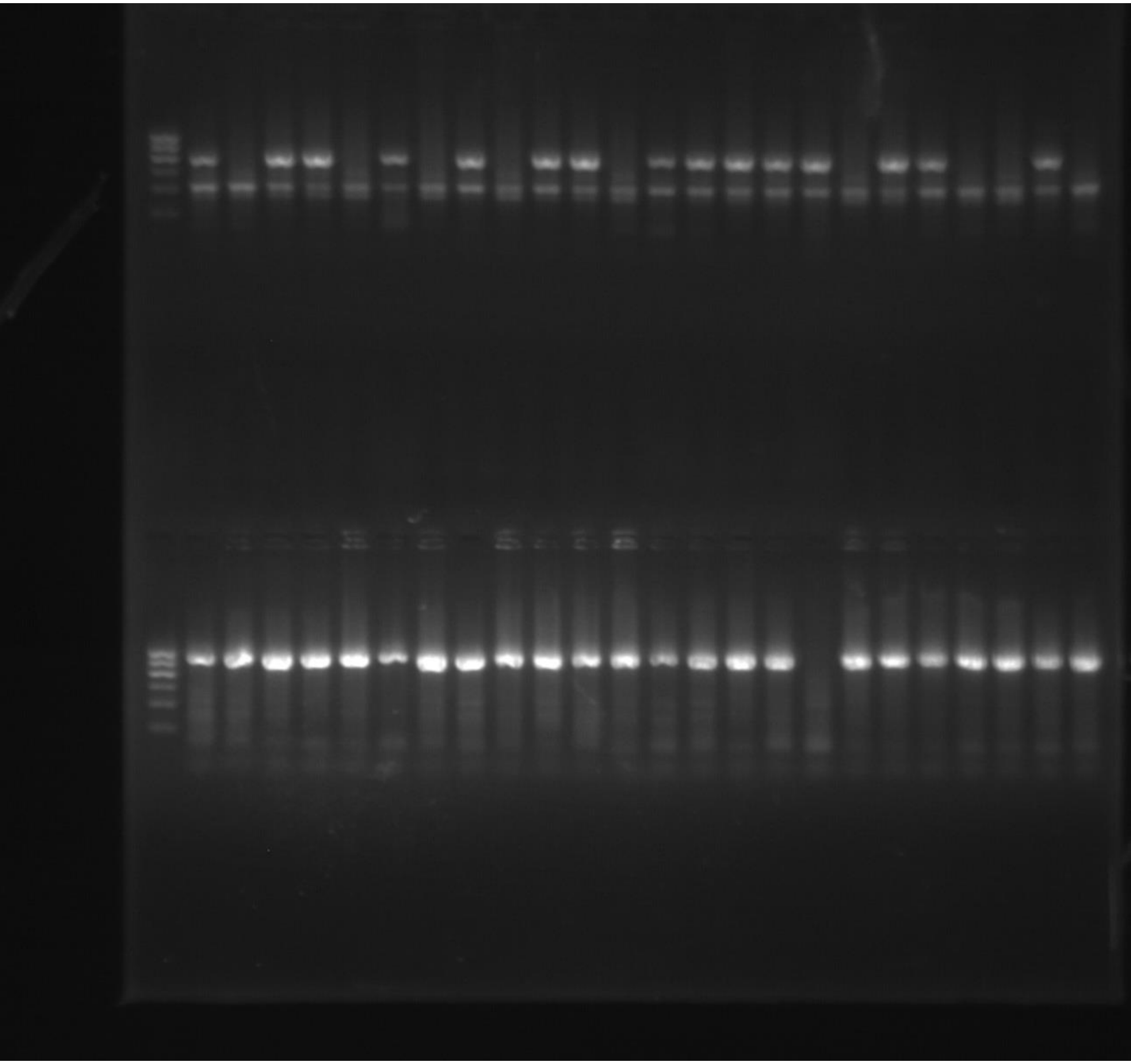

Source data of Suppl Fig 1C – original gel of CB1R

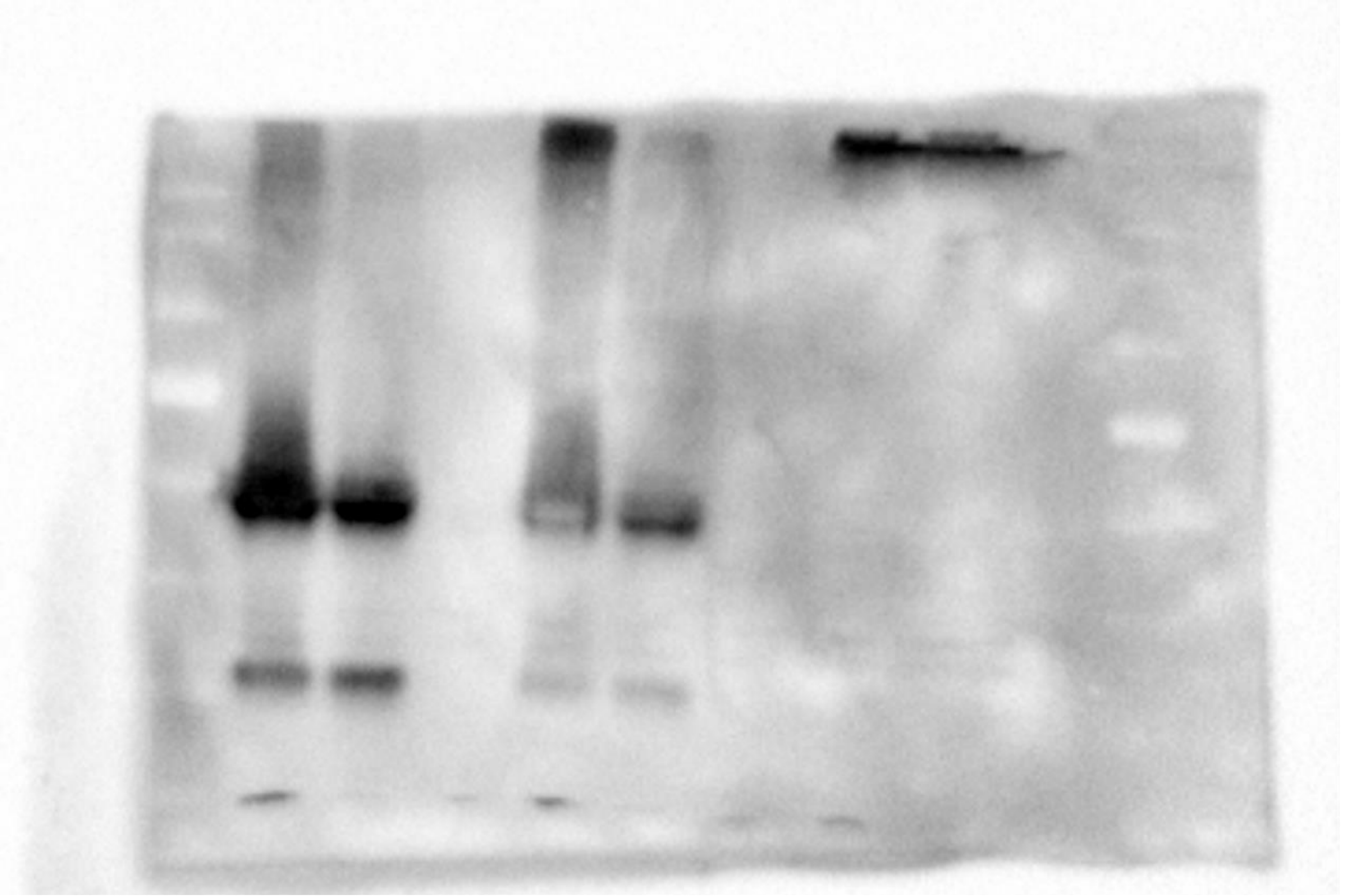

Source data of Suppl Fig 1C – original gel of GAPDH

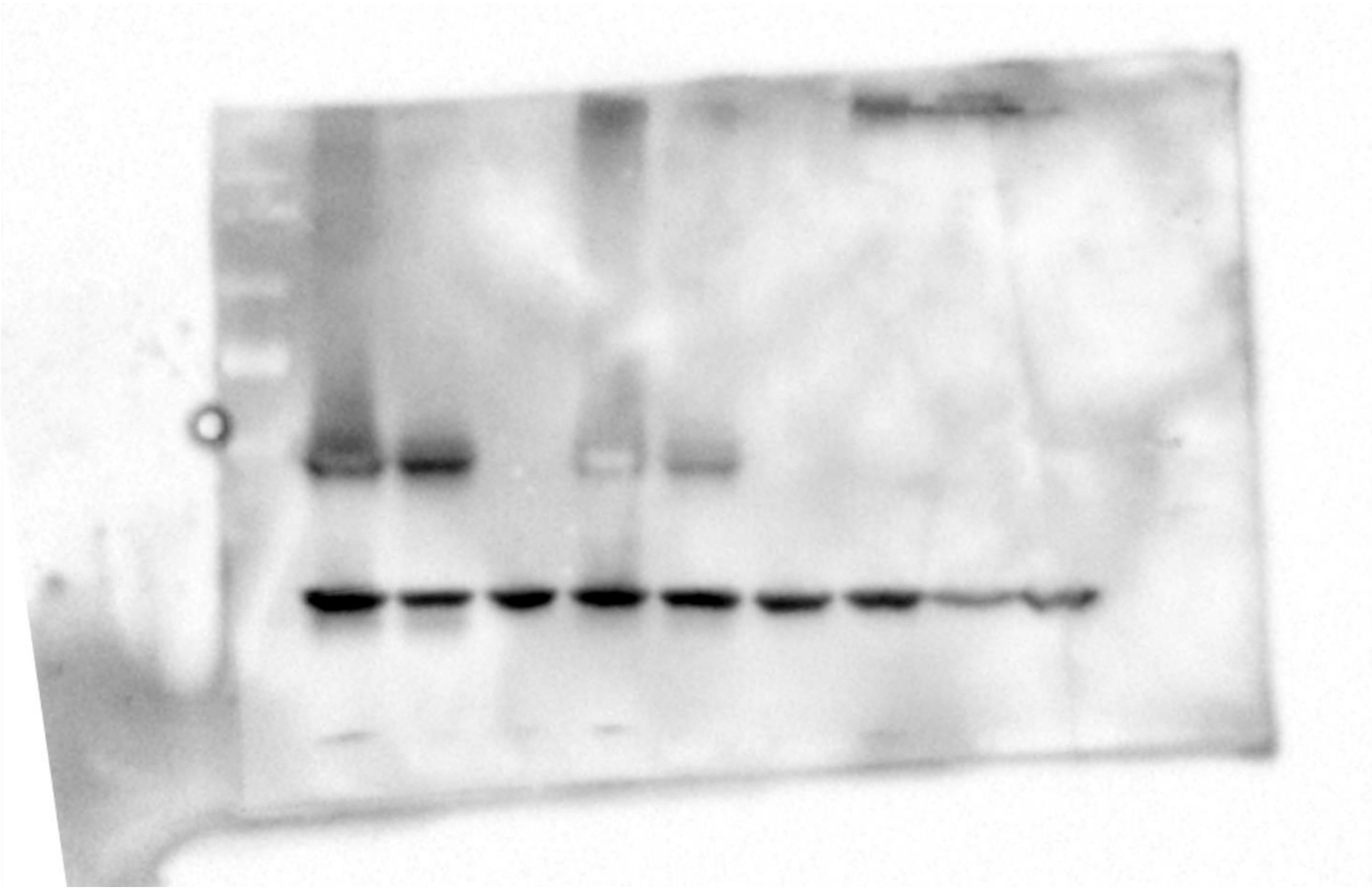

Source data of Suppl Fig 1D – original image of CB1R+/+ 60x

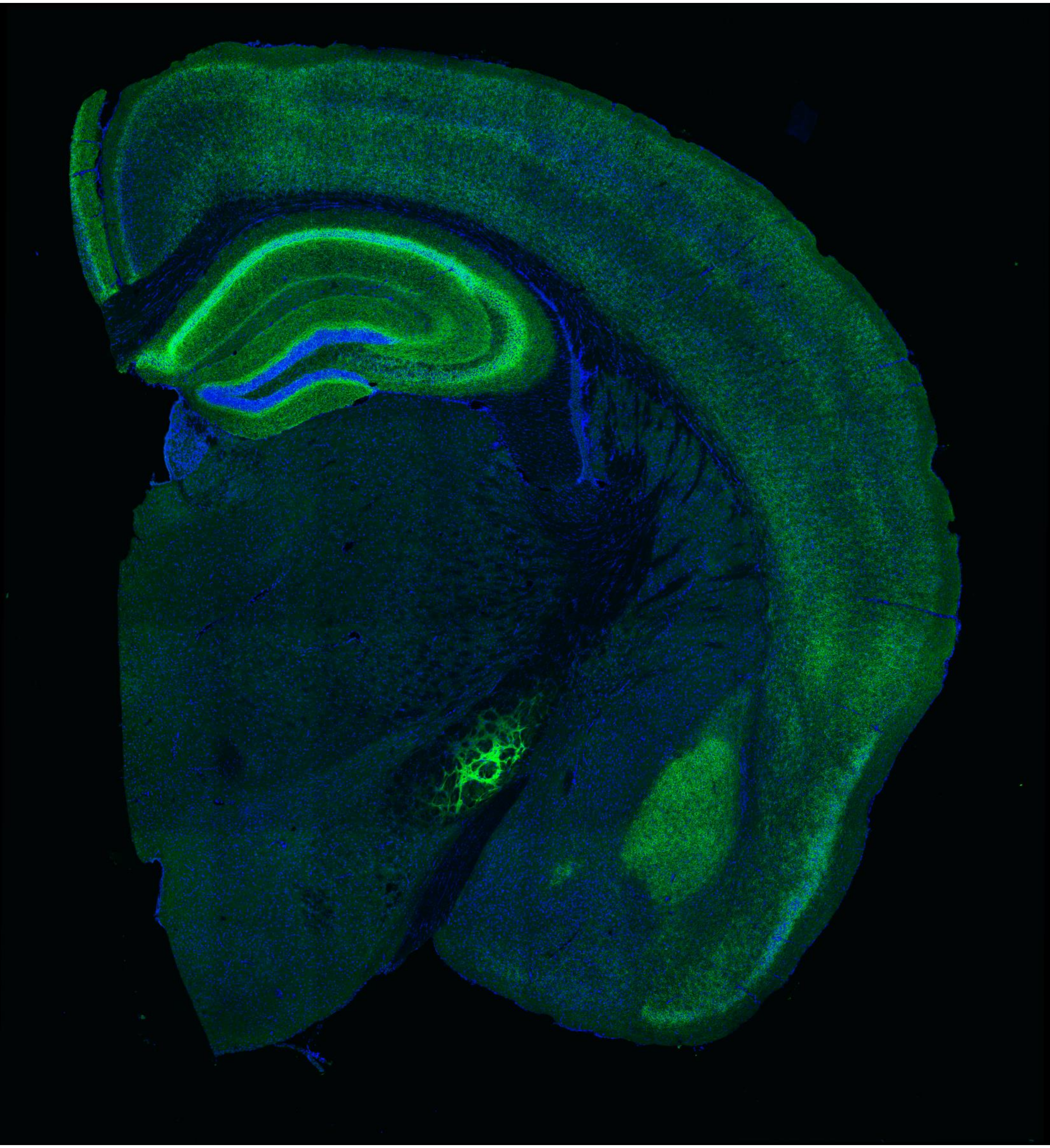

Source data of Suppl Fig 1D – original image of CB1R+/+ 20x

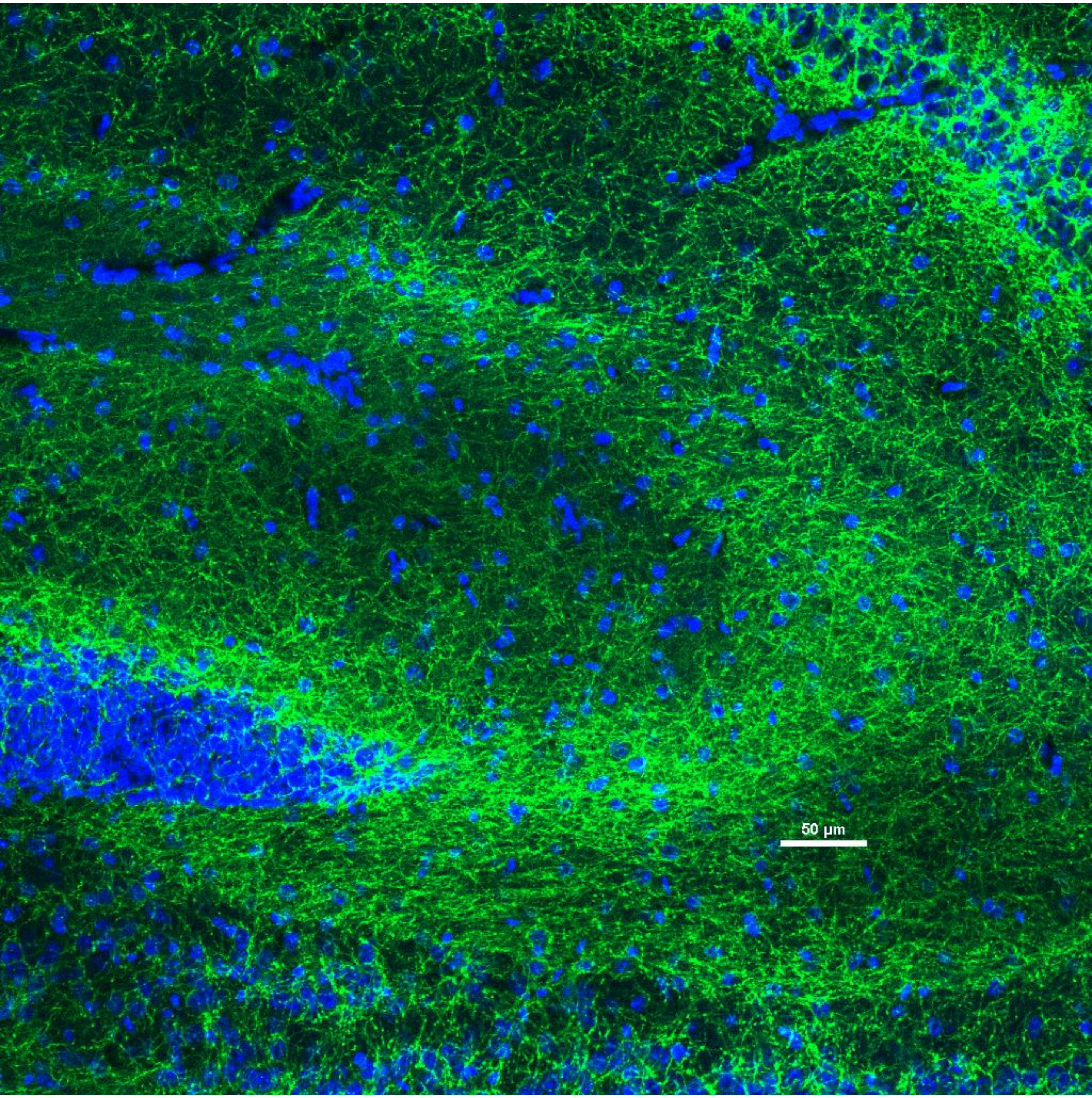

Source data of Suppl Fig 1D – original image of CB1R-/- 60x

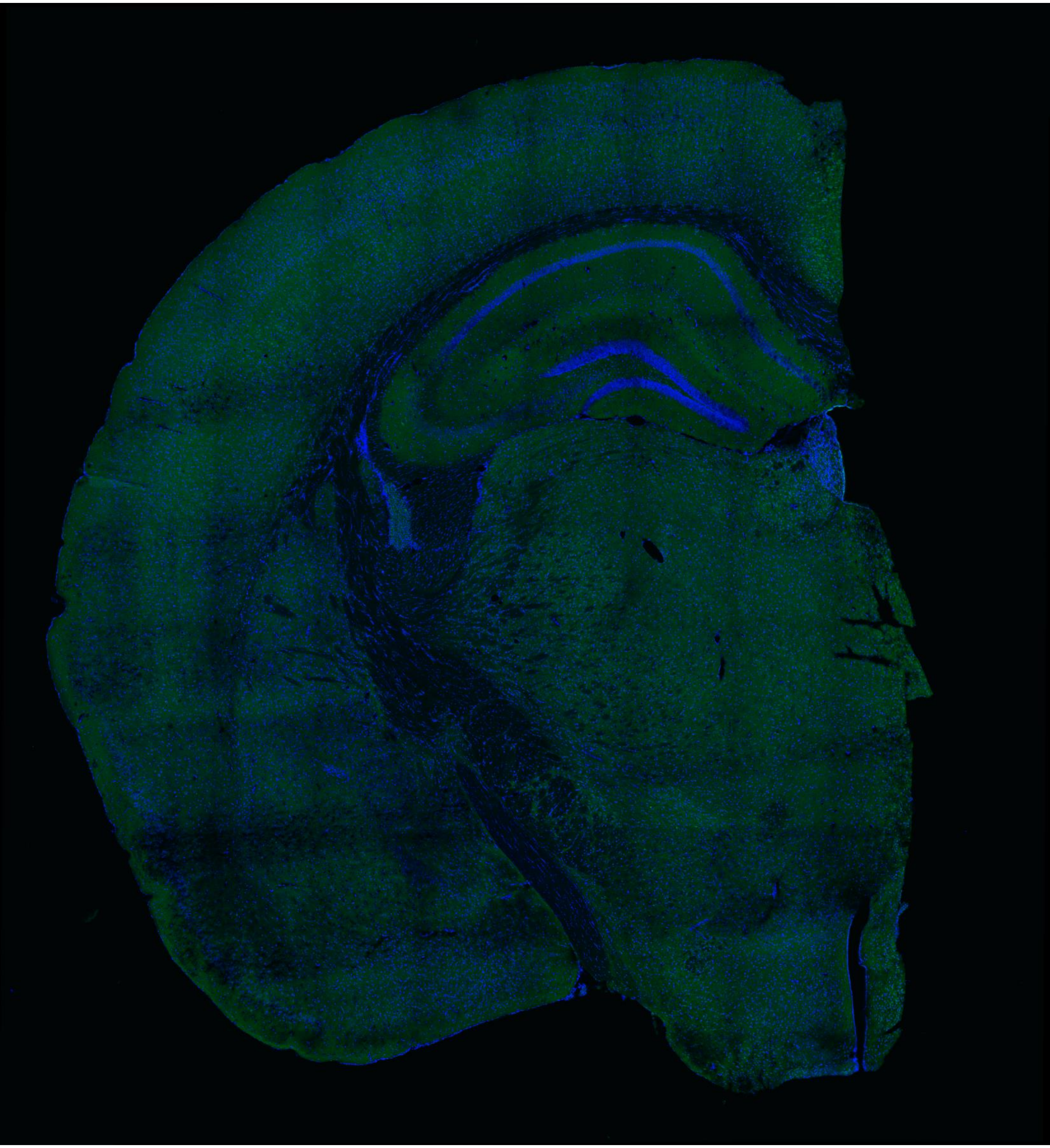

Source data of Suppl Fig 1D – original image of CB1R-/- 20x

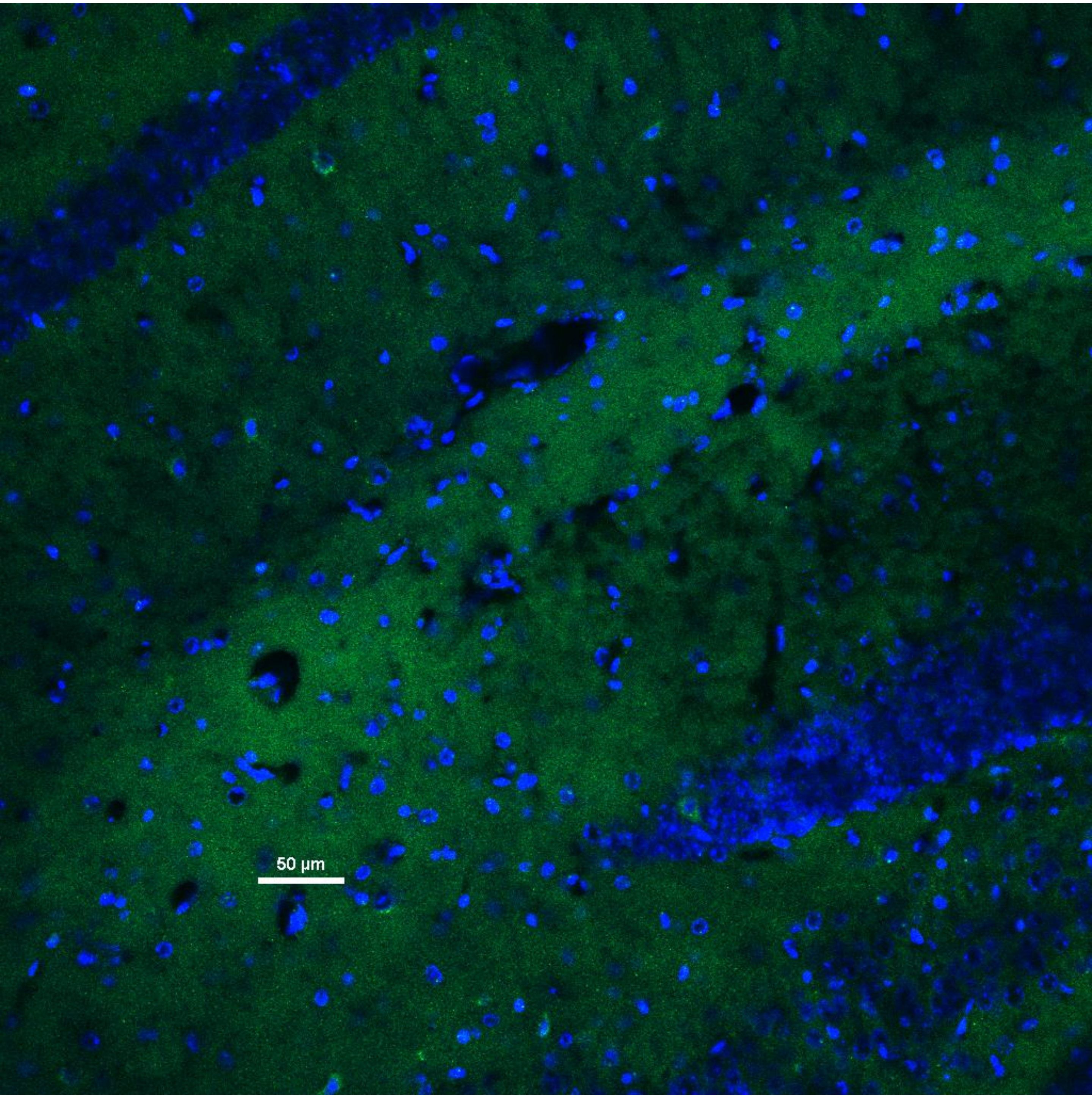

Supplement: Supplementary file 2 [file Data_Sheet_1.PDF]
